# Tolerance with High Yield Potential Is Provided by Lower Na^+^ Ion Accumulation and Higher Photosynthetic Activity in Tolerant YNU31-2-4 Rice Genotype under Salinity and Multiple Heat and Salinity Stress

**DOI:** 10.3390/plants12091910

**Published:** 2023-05-08

**Authors:** Lutfun Nahar, Murat Aycan, Ermelinda Maria Lopes Hornai, Marouane Baslam, Toshiaki Mitsui

**Affiliations:** 1 Department of Life and Food Science, Graduate School of Science and Technology, Niigata University, Niigata 950-2181, Japan; lutfun.sau@gmail.com (L.N.);; 2 Department of Agricultural Botany, Sher-e-Bangla Agricultural University, Dhaka 1207, Bangladesh; 3 JSPS International Research Fellow, Graduate School of Science and Technology, Niigata University, Niigata 950-2181, Japan; 4 Laboratory of Biochemistry, Faculty of Agriculture, Niigata University, Niigata 950-2181, Japan; 5 National Division of Research and Statistics, Timor-Leste Ministry of Agriculture and Fisheries, Dili 626, Timor-Leste; 6 Centre d’Agrobiotechnologie et Bioinge’ Nierie, Unite’ deRecherche labellise’ e CNRST (Centre AgroBio-tech-URL-CNRST-05), Universite’ Cadi Ayyad, Marrakech 40000, Morocco; 7 Laboratory of Agro-Food, Biotechnologies, and Valorization of PlantBioresources (AGROBIOVAL), Department of Biology, Faculty of Science Semlalia, Cadi Ayyad University (UCA), Marrakesh 40000, Morocco

**Keywords:** yield traits, high-temperature, abiotic stress, stress tolerance, morpho-physiological traits

## Abstract

The yield-reduction effect of abiotic stressors such as salinity and heat stresses with the growing world population threatens food security. Although adverse effects of salinity and heat stress on plant growth and production parameters have been documented, in nature, abiotic stresses occur sequentially or simultaneously. In this study, the stress tolerance and yield capacity of Yukinkomai, YNU31-2-4, and YNU SL rice genotypes tested under control (26 °C, 0 mM NaCl), salinity (26 °C, 75 mM NaCl), heat (31 °C, 0 mM NaCl), and heat and salinity (31 °C, 75 mM NaCl) stress combinations at vegetative and reproductive stages with six different scenarios. The results show that salinity and the heat and salinity combination stresses highly reduce plant growth performance and yield capacity. Heat stress during reproduction does not affect the yield but reduces the grain quality. The YNU31-2-4 genotype performs better under heavy salt and heat and salinity stress then the Yukinkomai and YNU SL genotypes. YNU31-2-4 genotypes accumulate less Na^+^ and more K^+^ under salt and multiple stresses. In the YNU31-2-4 genotype, low Na^+^ ion accumulation increases photosynthetic activity and pigment deposition, boosting the yield. Stress lowers the glucose accumulation in dry seeds, but the YNU31-2-4 genotype has a higher glucose accumulation.

## 1. Introduction

After receiving the first dangerous wave of global warming effects, environmental stressors have gained more importance relating to plant-yield potential. In particular, the yield reduction combined with the growing world population poses a danger to food security and creates a global alarm in agricultural associations. The issue of food security is of the highest priority due to the exponential increase in the global population, projected to reach 9 billion within the next three decades [1]. Presently, it is estimated that approximately 690 million people, which accounts for 11% of the global population, are confronted with hunger. Furthermore, projections indicate that the need for sustenance is expected to rise by 85%, equating to roughly 2.7 billion people, by the year 2050 [2,3]. Soil salinity and heat (high temperature) stresses become the most critical limiting factors to crop production worldwide [4]. Over 20% of the total global irrigated area is affected by high salinity [5,6]. High salt concentrations, affecting > 3% (397 Mha) of the total land area and crop productivity, cause losses of 55%, 28%, and 15% in corn, wheat, and cotton yield and US$ 27.3 billion a year [7]. By 2050, approximately 50% of the total arable agricultural land is expected to face high salinity problems [8]. Between 1880 and 2012, the average global combined land and ocean surface temperature increased by 0.85 degrees Celsius [9]. From here on, an average increase of at least 0.2 degrees Celsius every decade is predicted. The increasing concentration of greenhouse gases is a significant contributor to global warming. CO_2_ and methane concentrations have increased by 30 and 150% over the last 250 years [10,11]. These pressures have the most significant impact on plant development and productivity of any environmental element. For example, worldwide wheat production was predicted to fall by 6% for every degree Celsius increase in temperature [12]. Although rising temperatures benefit crop output in certain, more excellent, parts of the planet, the overall impact on global food security remains negative [13].

The detrimental impacts of salinity and heat stress on plant growth and production parameters have been extensively documented by numerous researchers [14,15,16,17,18]. However, under field conditions, plants may well be exposed to various abiotic stresses that occur sequentially or simultaneously during their lifespan. The responses of plants subjected to multiple stress combinations remain unknown [19]. The impact of a singular stressor on a plant’s response to various stressors may exhibit either complementary or antagonistic effects. This is because distinct stress factors influence the plant’s reaction to different stressors, even though individual stress variables control the plant’s response to other stressors [20]. Stresses that occur concurrently in the field may modify plant metabolism more precisely than different stress. In the face of concurrent heat and salt stress, plants exhibit a heightened K^+^ concentration and a diminished Na^+^/K^+^ ratio through the accumulation of glycine betaine and trehalose [21]. As a physiological activity, plants cannot sustain a high K^+^/Na^+^ ratio in their cytosol because salinity increases Na^+^ efflux [22], while heat stress decreases K^+^ efflux [23] in the roots. To adjust to such a stressful situation, crops endogenously develop compatible solutes (e.g., sugars, proline, carbohydrates, amino acids, phenolics, polyols, polyamines, or lipids) which have many different biological functions [24] and protective (e.g., heat shock proteins) proteins [25,26,27] to counteract oxidative stress. Upregulation of HKT, NHX, and SOS genes are just a few ways plants keep their Na^+^ and K^+^ levels stable. In addition, earlier research has shown that heat stress transcription factors (HSFs) regulate the HSP70 and HSP22 genes, which play critical roles in cell responses to heat stress [28]. Phytohormones such as ABA [29] and SA [30] mitigate the suppressive impact of salinity and high temperature through the regulation of biological and biochemical mechanisms associated with developmental processes.

Previously we showed rice genotypes’ tolerance capacity and mechanism under salinity, high-temperature stress, and stress release at the seedling stage [31]. However, plants may be exposed to various abiotic stress combinations that occur sequentially or simultaneously in natural habitats. In this study, we aim to test three rice genotypes under 6 different stress scenarios combinations of control (26 °C, 0 mM NaCl), salinity (26 °C, 75 mM NaCl), heat (31 °C, 0 mM NaCl), and heat and salinity (31 °C, 75 mM NaCl) stress at vegetative and reproductive stages. We determine yield and yield-related traits, leaf gas exchange traits, and morphologic and biochemical traits under single stress and stress combinations and evaluate all data by tolerance-related traits/markers by deep learning approaches to identify multiple stress tolerant genotypes.

## 2. Results

### 2.1. YNU31-2-4 Rice Genotype Shows Higher Growth Performance under Salinity and Heat and Salinity Stress

**Figure 1:**
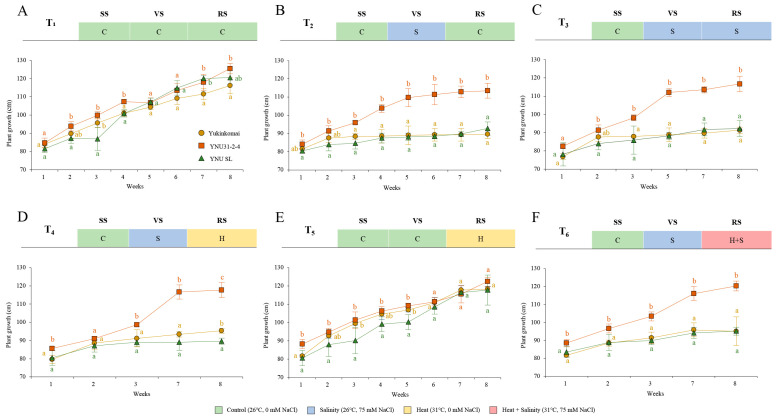
Weekly plant growth performance of Yukinkomai, YNU31-2-4, and YNU SL rice genotypes from the vegetative stage. Forty-day-old uniform-looking rice plants subjected to (**A**) treatment 1, (**B**) treatment 2 (T_2_), (**C**) treatment 3 (T_3_), (**D**) treatment 4 (T_4_), (**E**) treatment 5 (T_5_), and (**F**) treatment 6 (T_6_). Week 1 represents plant growth differences after 7 days of stress treatment at the vegetative stage. Seedling stage (SS), vegetative stage (VS), and reproductive stage (RS). The Tukey HSD test from three independent biological replicates (*n* = 5) shows that means (±SD) in the same graph followed by letters are substantially different at *p* < 0.05.

The plant growth performance of Yukinkomai, YNU31-2-4, and YNU SL rice genotypes from the vegetative stage under control, salinity, heat, and heat and salinity stress combinations (T_1_, T_2_, T_3_, T_4_, T_5_, and T_6_) are shown in Figure 1 and Figure S1. Under control (26 °C, 0 mM NaCl) conditions at the seedling stage (SS), vegetative stage (VS), and reproductive stage (RS), which is treatment 1 (T_1_), the YNU31-2-4 genotype shows a higher growth compared with the Yukinkomai genotype. Still, the YNU SL genotype does not show significantly different growth performance compared with the YNU31-2-4 and Yukinkomai genotypes at week 8 (W8) under control conditions (Figure 1A). Although uniform-looking plants were selected as beginning populations at the VS (Figure 1, 0 points of X axis), in the next 3 weeks, the YNU SL and Yukinkomai genotypes show different developmental responses under control conditions (Figure 1A,E). These differences may be welded genotypic and environmental conditions under control conditions. Treatment 2 (T_2_) represents salinity (26 °C, 75 mM NaCl) stress at only VS, and the YNU31-2-4 genotype shows significantly highest growth performance from W3 to W8 under T_2_ compared with the Yukinkomai and YNU SL rice genotypes (Figure 1B). Salinity stress at VS and RS was applied in treatment 3 (T_3_), and the YNU31-2-4 genotype shows a salinity tolerance as better plant growth compared with the Yukinkomai and YNU SL genotypes (Figure 1C). The rice genotypes were subjected to salinity at VS and heat (31 °C, 0 mM NaCl) at RS in treatment 4 (T_4_), and YNU31-2-4 shows the highest durability, followed by the Yukinkomai and YNU SL genotypes, respectively (Figure 1D). Treatment 5 (T_5_) represents heat stress at RS. Surprisingly all genotypes show almost similar growth patterns, and significantly different plant growth are not observed among all genotypes (Figure 1E). Treatment 6 (T_6_) represents salinity at VS and heat and salinity (31 °C, 75 mM NaCl) at RS as multiple stress conditions. The YNU31-2-4 rice genotype shows the highest growth performance not only with salinity stress at VS but also under heat and salinity stress at RS (Figure 1F).

### 2.2. YNU31-2-4 Rice Genotype Defense Photosynthetic Activity by Higher Chlorophyll Content and Stomatal Conductance from Heat and Salinity Stress Damage

**Figure 2:**
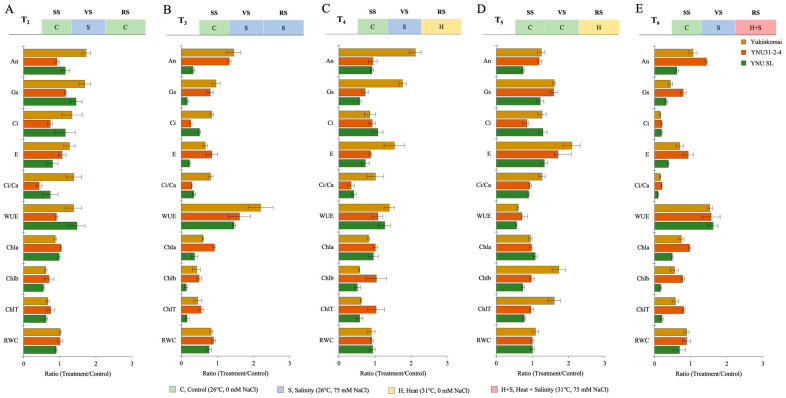
The ratio of photosynthetic parameters (The net photosynthetic rate, A_n_; stomatal conductance, g_s_; the intercellular CO_2_ concentration, C_i_; transpiration rate, E; the ratio of intercellular to ambient CO_2_ concentration, C_i_/C_a_; water use efficiency, WUE (A_n_/g_s_); chlorophyll a, Chla; chlorophyll b, Chlb; total chlorophyll, chlT content; and relative water content, RWC) of Yukinkomai, YNU31-2-4, and YNU SL rice genotypes under (**A**) treatment 2 (T_2_), (**B**) treatment 3 (T_3_), (**C**) treatment 4 (T_4_), (**D**) treatment 5 (T_5_), and (**E**) treatment 6 (T_6_). Ratio (Treatment/Control-T_1_). Seedling stage (SS), vegetative stage (VS), and reproductive stage (RS).

The salt stress at the VS (T_2_) significantly reduces Chlb and ChlT in all genotypes compared with the T_1_ condition, but the reduction percentage is lower in the YNU31-2-4 genotype. The T_2_ condition significantly increases A_n_ and g_s_ traits in all genotypes compared with the T_1_ condition. The highest A_n_ and g_s_ are found in the YNU SL genotype compared with the Yukinkomai and YNU31-2-4 genotypes (Figure 2A and Table A1 and Table A2). The salinity stress at the VS and RS (T_3_) significantly reduces all traits except A_n_ in Yukinkomai and YNU31-2-4 genotypes and WUE traits. The highest A_n_, g_s_, E, Chla, Chlb, ChlT, and RWC are found in the YNU31-2-4 genotype compared with the Yukinkomai and YNU SL genotypes (Figure 2B and Table A1 and Table A2). The salinity and heat stress at the VS and RS (T_4_) increases the WUE in Yukinkomai (39%), YNU31-2-4 (7%), and YNU SL (26%) genotypes. The highest C_i_, Chla, Chlb, ChlT, and RWC traits are found in the YNU31-2-4 genotype. The highest A_n_ and g_s_ are found in the Yukinkomai genotype, while it is significantly decreased in the YNU31-2-4 and YNU SL genotypes (Figure 2C and Table A1 and Table A2). The heat stress at the RS (T_5_) significantly increases g_s_ and E compared with T_1_ in all genotypes. The highest An and WUE are detected in the YNU31-2-4 genotype. Furthermore, T_5_ significantly reduces the Chla content in all genotypes, but the reduction percentage is lower in the YNU31-2-4 genotype compared with the Yukinkomai and YNU SL genotypes (Figure 2D and Table A1 and Table A2). The salinity at VS and heat and salinity at RS (T_6_) significantly reduce g_s_, C_i_, E, C_i_/C_a_, Chla, Chlb, ChlT, and RWC in all genotypes, but the lowest reduction is recorded in the YNU31-2-4 genotype compared with the Yukinkomai and YNU SL genotypes. The highest A_n_, g_s_, C_i_, C_i_/C_a_, Chla, Chlb, ChlT, and RWC are observed in the YNU31-2-4 genotype (Figure 2E and Table A1 and Table A2 and Table S1).

### 2.3. YNU31-2-4 Genotype Shows Higher Plant Growth and Yield Performance under Salt, Heat, and Heat + Salt Stress Treatments

**Figure 3:**
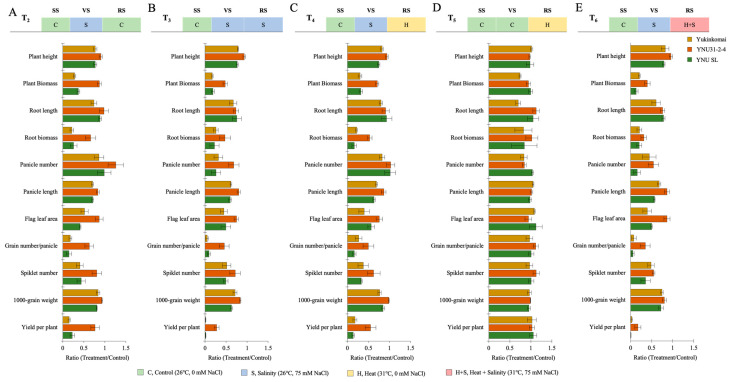
The ratio of harvesting parameters (plant height, plant biomass, root length, root biomass, panicle number, panicle length, flag leaf area, grain number per panicle, spikelet number, 1000-grain weight, and yield per plant) of Yukinkomai, YNU31-2-4, and YNU SL rice genotypes under (**A**) treatment 2 (T_2_), (**B**) treatment 3 (T_3_), (**C**) treatment 4 (T_4_), (**D**) treatment 5 (T_5_), and (**E**) treatment 6 (T_6_). Ratio (Treatment/Control-T_1_). Seedling stage (SS), vegetative stage (VS), and reproductive stage (RS).

The salt stress at the VS (T_2_) significantly reduces plant height (PH), plant biomass (PB), root length (RL), root biomass (RB), root length (RL), root biomass (RB), panicle number (PN), panicle length (PL), flag leaf area (FLA), grain number per panicle (GPP), spikelet number (SN), 100-grain weight (TGW), and yield per plant (YPP) in all genotypes, except the panicle number of YNU31-2-4 genotype is significantly increased by 22% compared with T_1_. Although reductions are observed, less reduction is recorded in the YNU31-2-4 genotype under T_2_ compared with T_1_ conditions (Figure 3A and Table A3 and Table A4). The salinity stress at the VS and RS (T_3_) significantly reduce all measured traits; in particular, the plant biomass of Yukinkomai, YNU31-2-4, and YNU SL genotypes are decreased by 82, 52, and 81% under T_3_ compared with T_1_ conditions. The YNU31-2-4 genotype reduces all measured traits less than the Yukinkomai and YNU SL genotypes under T_3_ conditions (Figure 3B and Table A3 and Table A4). The salinity and heat stress at the VS and RS (T_4_) reduces all measured traits. The PB and RB are highly reduced by approximately 68 to 83% in the Yukinkomai and YNU SL genotypes, but it is reduced by 29 and 47% in the YNU31-2-4 genotype, respectively. The highest PH, PB, RB, PL, FLA, GNPP, SN, TGW, and YPP were found in the YNU31-2-4 rice genotype under the T_4_ condition (Figure 3C and Table A3 and Table A4). The heat stress at the RS (T_5_) negatively affects measured traits less than T_2_, T_3_, T_4_, and T_6_ conditions. The YNU31-2-4 genotype shows significantly higher RL, RB, and TGW under T_5_ conditions. The other measured traits show no significant differences among the Yukinkomai, YNU31-2-4, and YNU SL genotypes under the T_5_ condition (Figure 3D and Table A3 and Table A4). The salinity at VS and heat and salinity at RS (T_6_) are significantly reduced in all measured traits compared with T_1_. The YNU31-2-4 genotype shows the lowest reduction in PH, PB, RB, PN, PL, FLA, GNPP, SN, TGW, and YPP traits, and it shows the highest PH, PB, PL, FLA, GNPP, TGW, and YPP compared with T_1_ under T_6_ condition (Figure 3E and Table A3 and Table A4).

### 2.4. YNU31-2-4 Genotype Has High Perfect Grain Potential under Salinity and Heat and Salinity Stress Treatments

**Figure 4:**
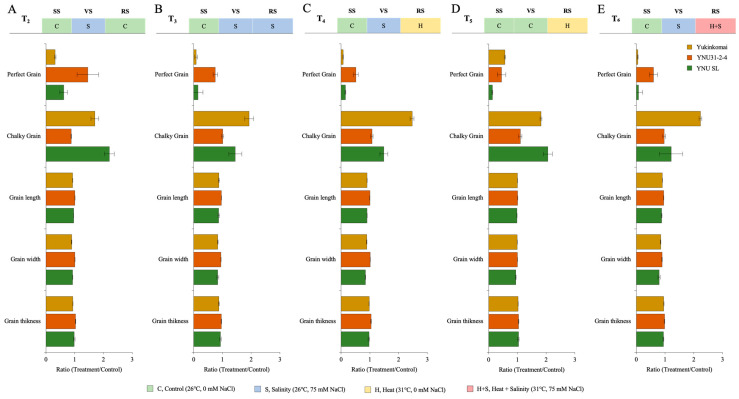
The ratio of grain quality parameters (Grain length, grain width, grain thickness, perfect grain, and chalky grain) of Yukinkomai, YNU31-2-4, and YNU SL rice genotypes under (**A**) treatment 2 (T_2_), (**B**) treatment 3 (T_3_), (**C**) treatment 4 (T_4_), (**D**) treatment 5 (T_5_), and (**E**) treatment 6 (T_6_). Ratio (Treatment/Control-T_1_). Seedling stage (SS), vegetative stage (VS), and reproductive stage (RS).

The grain size and quality parameters, such as grain length (GL), grain width (GW), grain thickness (GT), and perfect grain (PG) numbers, are significantly reduced under all stress treatments. Still, the number of chalky grains (CG) significantly increases. The salinity at VS (T_2_) significantly decreases grain size (GL, GW, and GT) and PG number in the Yukinkomai and YNU SL genotypes. Still, the YNU31-2-4 genotype increases the PG number by 45% and reduces the CG number by 12% compared with T_1_ under the T_2_ condition (Figure 4A and Table A5). The salinity stress at VS and RS (T_3_) significantly reduces grain size and PG number. Surprisingly, the PG number decreases by 91 and 85% in Yukinkomai and YNU SL genotypes, and the PG number of YNU31-2-4 is only reduced by 24% compared with T_1_ under the T_3_ condition. The CG number is increased in Yukinkomai (92%) and YNU SL (44%) genotypes but almost not changed in YNU31-2-4 (0.6%) genotypes. The grain size is also significantly reduced in all genotypes, but the reduction percentage is lower in the YNU31-2-4 rice genotype than in T_1_ under the T_3_ condition (Figure 4B and Table A5). A similar pattern is also observed under the T_4_ condition. The YNU31-2-4 genotype only has a 24% reduction in the PG number, but the Yukinkomai and YNU SL genotypes have a 91 and 85% reduction in PG number compared with T_1_ under the T_4_ condition, respectively. The CG number is significantly increased by 146 and 48% in the Yukinkomai and YNU SL genotypes, but the CG number of the YNU31-2-4 genotype just increases by 7% compared with T_1_ under the T_4_ condition. Furthermore, less grain size reduction is recorded in the YNU31-2-4 genotype compared with the Yukinkomai and YNU SL genotypes under the T_4_ condition (Figure 4C and Table A5). The heat stress at RS negatively affects the PG number in all genotypes, but the Yukinkomai genotype has a lower reduction than the YNU31-2-4 and YNU SL genotypes under the T_5_ condition. The CG number is found to increase in all genotypes. Still, an increment in the percentage of YNU31-2-4 is detected as lower than in Yukinkomai and YNU SL genotypes under the T_5_ condition. The heat stress increases the GT number in all genotypes, and the highest increment is observed in the YNU31-2-4 genotype under the T_5_ condition (Figure 4D and Table A5). The salinity at VS and heat and salinity stress at RS significantly reduce the PG number by 95 and 92% in Yukinkomai and YNU SL genotypes. Still, it reduces the PG number by 40% in the YNU31-2-4 genotype under the T_6_ condition. The CG number significantly increases by 123 and 21% in Yukinkomai and YNU SL genotypes, and it is raised by just 3% in the YNU31-2-4 genotype under the T_6_ condition. The T_6_ significantly reduces grain size, but the reduction rate is lower in the YNU31-2-4 genotype than in the Yukinkomai and YNU SL genotypes under the T_6_ condition (Figure 4E and Table A5).

### 2.5. YNU31-2-4 Genotype Increased CAT Activity under Stress Conditions

**Figure 5:**
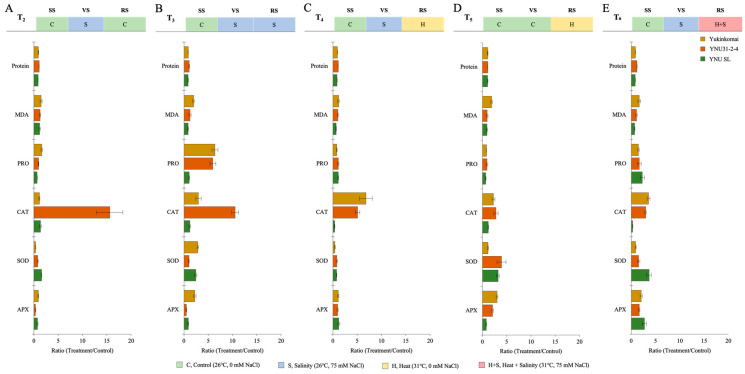
The ratio of protein, malondialdehyde (MDA), proline (PRO) content, catalase (CAT), superoxide dismutase (SOD), and ascorbate peroxidase (APX) activity of Yukinkomai, YNU31-2-4, and YNU SL rice genotypes under (**A**) treatment 2 (T_2_), (**B**) treatment 3 (T_3_), (**C**) treatment 4 (T_4_), (**D**) treatment 5 (T_5_), and (**E**) treatment 6 (T_6_). Ratio (Treatment/Control-T_1_). Seedling stage (SS), vegetative stage (VS), and reproductive stage (RS).

The salinity at VS (T_2_) shows a significant reduction of protein (PROT), proline (PRO) content, superoxide dismutase (SOD) partly, and ascorbate peroxidase (APX) activity in all genotypes. The malondialdehyde (MDA) content is significantly increased in all genotypes, but lower MDA content and higher PRO and APX content are recorded in the YNU SL genotype under T_2_. The CAT activity of the YNU31-2-4 genotype is significantly increased compared with the control (T_1_) under the T_2_ condition (Figure 5A and Table A6). The salinity at VS and RS (T_3_) reduces the protein content in Yukinkomai and YNU SL genotypes but increases in the YNU31-2-4 genotype under the T_3_ condition. The highest MDA, PRO content, SOD, and APX activity is observed in the Yukinkomai genotype. YNU31-2-4 genotype shows higher CAT activity under the T_3_ condition (Figure 5B and Table A6). The salinity at VS and heat at RS (T4) reduce SOD activity in all genotypes. Still, less reduction and higher production are observed in the YNU31-2-4 genotype compared with the Yukinkomai and YNU SL genotypes under T_4_. Furthermore, PRO and SOD reduction is lower in the YNU31-2-4 genotype than in other tested genotypes under the T_4_ condition (Figure 5C and Table A6). The heat stress at RS (T_5_) reduces the PRO accumulation and increases PROT, CAT, SOD, and APX activity in all genotypes. The YNU31-2-4 genotype shows less PRO reduction than the Yukinkomai and YNU SL genotypes under the T_5_ condition. Furthermore, the YNU31-2-4 genotype shows a higher increment by 178, and 278% in CAT and SOD activity under the T_5_ condition, respectively (Figure 5D and Table A6). The salinity at VS and heat and salinity at RS (T6) increase PROT, PRO, CAT, SOD, and APX by 16, 63, 195, 52, and 64% in the YNU31-2-4 genotype, respectively (Figure 5E and Table A6).

### 2.6. YNU31-2-4 Genotype Has Lower Na^+^ Ion Accumulation under Salinity Stress Conditions

**Figure 6:**
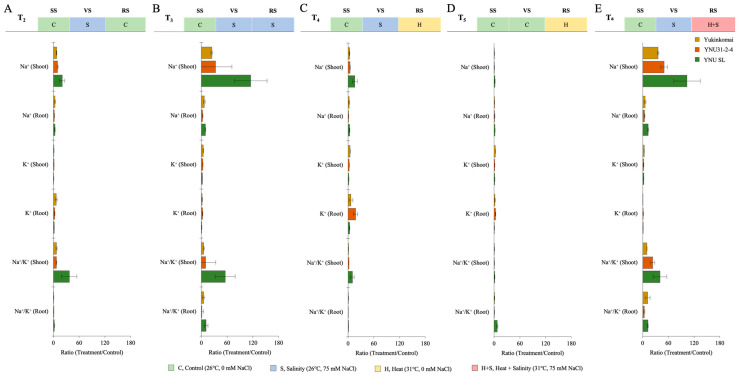
The ratio of ion content (root and shoot) of Yukinkomai, YNU31-2-4, and YNU SL rice genotypes under (**A**) treatment 2 (T_2_), (**B**) treatment 3 (T_3_), (**C**) treatment 4 (T_4_), (**D**) treatment 5 (T_5_), and (**E**) treatment 6 (T_6_). Ratio (Treatment/Control-T_1_). Seedling stage (SS), vegetative stage (VS), and reproductive stage (RS).

The salinity stress as VS (T_2_) increases ion accumulation in all genotypes compared with the T_1_ condition. The genotypes of Yukinkomai and YNU SL exhibit the greatest accumulation of Na^+^ ions in their shoots and roots. The YNU31-2-4 genotype exhibits reduced levels of shoot and root Na^+^ accumulation compared to the Yukinkomai and YNU SL genotypes when subjected to T_2_ conditions. The genotype YNU31-2-4 exhibits the greatest accumulation of K^+^ in shoot tissues during T_2_. Furthermore, it is observed that the YNU31-2-4 genotype exhibits a decreased Na^+^/K^+^ ratio when subjected to T_2_ (Figure 6A and Table A7). The salinity stress at VS and RS (T_3_) increases Na^+^ accumulation in the Yukinkomai, YNU31-2-4, and YNU SL genotypes compared with T_1_ and T_2_ conditions. Under the T_2_ condition, the highest Na^+^ accumulation at the shoot and root tissues is recorded in the YNU SL genotype, and the lowest Na^+^ accumulation is observed in the YNU31-2-4 genotype. 

The highest K^+^ accumulation at the shoot and root tissues is recorded in the YNU31-2-4 genotype under the T_3_ condition. The lowest Na^+^/K^+^ ratio is also observed in the YNU31-2-4 genotype (Figure 6B and Table A7). The salinity stress at VS and heat stress at RS (T_4_) significantly reduce Na^+^ ion accumulation at the shoot and root tissues in all genotypes compared with the control (T_1_) treatment. The lowest Na^+^ accumulation at the shoot and root tissues is observed in the YNU31-2-4 genotype compared with Yukinkomai and YNU SL genotypes. The highest K^+^ ion accumulation at the shoot and root tissues is recorded in YNU31-2-4 genotype under T_4_. The Na^+^/K^+^ ratio is also lower in YNU31-2-4 genotype under T_2_ (Figure 6C and Table A7). The ion accumulation of all genotypes is found to be very low under heat stress at RS (T_5_). The K^+^ ion accumulation of the Yukinkomai genotype at shoot tissues is increased by 172% compared with the control (T_1_) condition, and K^+^ accumulation of the YNU31-2-4 genotype at root tissues is increased by 198% compared with the control (T_1_) condition under T_5_ condition (Figure 6D and Table A7). The salinity stress at VS and heat and salinity stress at RS (T_6_) significantly increases Na^+^ ion accumulation compared with the control (T_1_) condition. The lowest Na^+^ ion accumulation and Na^+^/K^+^ ratio at the shoot and root tissues are observed in the YNU31-2-4 genotype under the T_6_ condition. Surprisingly, a higher K^+^ accumulation is observed in the YNU SL genotype under the T_6_ condition (Figure 6E and Table A7).

### 2.7. Salinity Reduces Glucose Accumulation in Dry Seeds, but the YNU31-2-4 Genotype Has Higher Glucose Accumulation under Salinity and Heat and Salinity Stress

**Figure 7:**
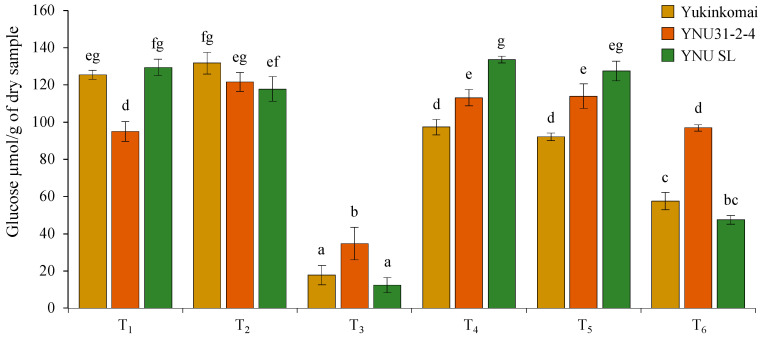
The glucose content of dry seeds of Yukinkomai, YNU31-2-4, and YNU SL rice genotypes under Treatment 1 (T_1_), T_2_, T_3_, T_4_, T_5_, and T_6_. The Tukey HSD test from three independent biological replicates (*n* = 3) shows that means (±SD) in the same graph followed by letters are substantially different at *p* < 0.05.

Under control (T_1_) conditions, the YNU31-2-4 genotype has lower glucose (GLU) content in dry seeds compared with Yukinkomai and YNU SL genotypes. It is clear that the salinity stress at VS (T_2_) does not affect the GLU accumulation of the Yukinkomai and YNU SL genotypes, but it increases the GLU accumulation by 27% in the YNU31-2-4 genotype. The salinity stress at VS and RS (T_3_) significantly reduces the GLU accumulation by 85, 63, and 90% in dry seeds of Yukinkomai, YNU31-2-4, and YNU SL genotypes. The highest GLU accumulation is recorded in the YNU31-2-4 genotype under the T_3_ condition. The salinity stress at VS and heat stress at RS (T_4_) decrease GLU accumulation by 22% in the Yukinkomai genotype but significantly increase GLU accumulation by 19 and 3% in YNU31-2-4 and YNU SL genotypes compared with control (T_1_) condition, respectively. The heat stress at RS (T_5_) significantly reduces the GLU accumulation by 26 in the Yukinkomai genotype, but it significantly increases the GLU accumulation by 20% in the YNU31-2-4 genotype. Lastly, the salinity stress at VS and heat and salinity stress at RS (T_6_) significantly reduce GLU accumulation by 54 and 63% in Yukinkomai and YNU SL genotypes compared with control (T_1_) conditions. Still, they do not change the GLU accumulation in the YNU31-2-4 genotype (Figure 7).

### 2.8. YNU31-2-4 Genotype Showed Higher Salinity Stress and Heat and Salinity Stress Tolerance according to the Overall Result Evaluation by PCA and HCA Clustering

**Figure 8:**
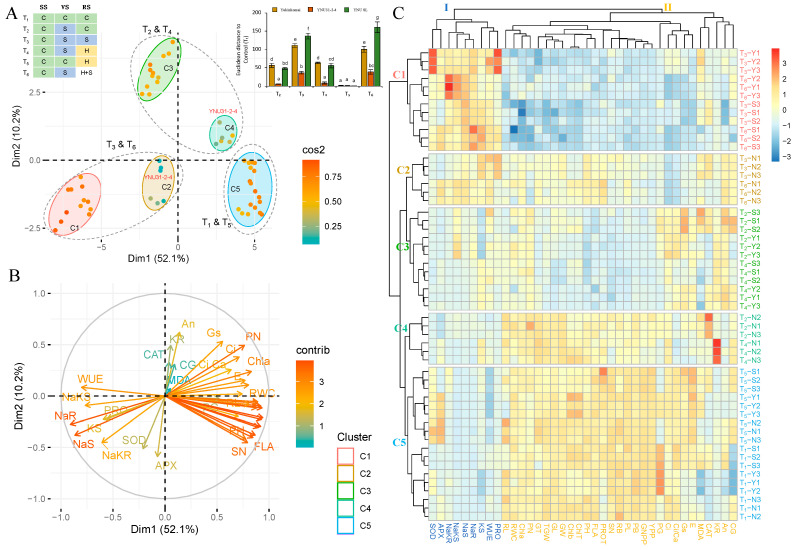
Principle component and hierarchical clustering analyses of growth performance of Yukinkomai, YNU31-2-4, and YNU SL rice genotypes under different salt and heat stress combinations (T_1_, T_2_, T_3_, T_4_, T_5_, and T_6_) in different growth stages (SS: Seedling stage, VS: Vegetative stage, RS: Reproductive stage). (**A**) Principal component analysis (PCA) of the spatialization of genotypes and treatments (Colors green: control (C, 26 °C, 0 mM NaCl), blue: salinity (S, 26 °C, 75 mM NaCl), yellow: heat (H, 31 °C, 0 mM NaCl) and red: heat + salt (H + S, 31 °C, 75 mM NaCl) treatments), (**B**) PCA of the studied traits, and (**C**) hierarchical clustering analysis (HCA) of measured growth performance in Yukinkomai, YNU31-2-4, and YNU SL genotypes under treatment 1 (T1), T_2_, T_3_, T_4_, T_5_, and T_6_. Clusters represent genotypes (C1 to C7) and traits (I and II). Yukinkomai (Y), YNU31-2-4 (N), and YNU SL (S) genotypes. The net photosynthetic rate (An), stomatal conductance (Gs), the intercellular CO_2_ concentration (Ci), transpiration rate (E), the ratio of intercellular to ambient CO_2_ concentration (Ci/Ca), water use efficiency (WUE), chlorophyll a (Chla), chlorophyll b (Chlb), total chlorophyll (ChlT) content, and relative water content (RWC), plant height (PH), plant biomass (PB), root length (RL), root bimass (RB), panicle number (PN), panicle length (PL), flag leaf area (FLA), grain number perpanicle (GNPP), spiklet number (SN), 1000-grain weight (TGW), yield per plant (YPP), perfect grain (PG), chalky grain (CG), grain length (GL), grain width (GW), grain thickness (GT), protein (PROT), malondialdehyde (MDA), proline (PRO), catalase (CAT), superoxide dismutase (SOD), ascorbat peroxidase (APX), Na^+^ concentration in the shoot (NaS), Na^+^ concentration in the root (NaR), K^+^ concentration in the shoot (KR), K^+^ concentration in the root (KR), Na^+^/K^+^ ratio in the shoot (NaKS), and Na^+^/K^+^ ratio in the root (NaKR). The Tukey HSD test from three independent biological replicates (*n* = 3) shows that means (±SD) in the same graph followed by letters are substantially different at *p* < 0.05.

The principal component analysis (PCA) was performed on measured traits from three rice genotypes under six treatments (T_1_ to T_6_) to evaluate the salinity, heat, and heat and salinity stress tolerance capacity of genotypes and identify response similarity among treatments. The PCA showcases the performance of rice genotypes across various stress conditions. The analysis reveals two significant variables, namely Dimension 1 (Dim1) and Dim2, with Dim1 accounting for the majority share of 52.1% and Dim2 contributing 10.2%. In total, Dim1 and Dim2 together account for 62.3% of the observed variance (Figure 8A and Table S2). The hues of the distinct variables denote their level of representation quality of the principal component, which is abbreviated as ‘Cos2’ (Table S2). The Yukinkomai, YNU31-2-4, and YNU SL genotypes are clearly separate from each other based on the stress treatment in Figure 8A. The T_1_ and T_5_, T_2_ and T_4_, and T_3_ and T_6_ conditions are grouped, but the YNU31-2-4 genotypes group differently from the Yukinkomai and YNU SL genotypes under T_2_ and T_4_ and T_3_ and T_6_ conditions. The analysis of Euclidean distance, which depicts the correlation between rice germplasms cultivated under stress conditions, provides evidence for the differentiation between germplasms with higher and lower stress tolerance. (Figure 8A, right-top panel). Statistically significant differences (*p* < 0.05) are observed in the Euclidean distance between T_1_ and T_6_, with the YNU SL genotype showing the highest distance, followed by Yukinkomai. Conversely, the YNU31-2-4 genotype exhibits the lowest distance under T_2_, T_3_, T_4_, and T_6_ conditions. The data presented indicate the presence of elements in the Euclidean distance matrix of the tested accessions, which validate the YNU31-2-4 genotype as having significantly distinct responses to stress combinations T_2_, T_3_, T_4_, and T_6_. The WUE, PRO, SOD, APX, NaS, NaR, KS, NaKS, and NaKR traits are associated with the T_3_ and T_6_ conditions. CAT, KR, An, CG, MDA, Gs, PN, Ci, Ci, Ca, Chla, E, RL, and RWC traits are associated with the T_2_ and T_4_ conditions, and the rest of the traits are detected associated with the T_1_ and T_5_ conditions (Figure 8B).

The results of the two-way hierarchical clustering analysis (HCA) indicate that the measured traits observed under T_1_ to T_6_ conditions can be grouped into two distinct primary clusters, as depicted in the generated heat map (Figure 8C, groups I and II). Group I (PRO, WUE, KS, NaR, NaS, NaKS, NaKR, APX, and SOD) traits are highly expressed in the T_3_ and T_6_ conditions in all genotypes. Group II traits are highly expressed in T_1_ and T_5_ traits in all genotypes. The heatmap categorizes the Yukinkomai, YNU31-2-4, and YNU SL genotypes into five clusters using data from the T_1_ to T_6_ conditions (Figure 8C, groups C1 to C5). Under T_3_ and T_6_ conditions, Cluster 1 (C1) indicates Yukinkomai (Y) and YNU SLU (S) genotypes. The Yukinkomai genotype shows a high sensitivity to salinity and heat and salinity stress. The YNU31-2-4 (N) genotypes under T_3_ and T_6_ conditions are both assigned to C2. The Yukinkomai and YNU SL genotypes under T_2_ and T_4_ conditions regrouped in C3. Under the same condition (T_2_ and T_4_), the YNU31-2-4 genotype is separated from other genotypes and located in C4. Lastly, C5 represents the Yukinkomai, YNU31-2-4, and YNU SL genotypes under T_1_ and T_5_ conditions (Figure 8C).

## 3. Discussion

Plant growth is vulnerable to unfavorable climatic conditions, and agricultural systems take significant damage if there are not enough precautions to adapt plants to the changing environment [32]. Salinity, heat, drought, pollution, and soil nutrient deficiency are the main environmental limitations for modern agricultural applications. These abiotic stress factors compromise plant growth and development through morphological, physiological, biochemical, and molecular processes and result in yield reduction, in which animals and humans may face hunger soon [33]. The current approaches to evaluating the stress tolerance capacity of plants are based on single stress factor effects. Still, in natural habitats, plants may be exposed to various abiotic stress combinations that occur sequentially or simultaneously [20]. Our stress scenarios (T_1_, T_3_, T_5_, and T_6_) are modeled based on natural events, but two (T_3_ and T_4_) are hypothetical. In this experiment, plants are not exposed to any stress at the seedling stage (T_1_) because of two reasons: firstly, we tested the seedling stage multiple stress tolerance levels of rice genotypes in our previous study [31], and secondly, rice seedlings generally are planted in paddy fields at around 15–30 days old [34], so plants may not be exposed to stress factors during the seedling stage. In agriculture, soil salinity is typically assessed based on the electrical conductivity of the saturation extract (ECe), with a threshold of 4 deci-Siemens per meter (dSm^−1^) commonly used to define saline soils. Rice is very sensitive to salinity. Field studies have shown that a seasonal salinity of the field water over 1.9 dSm^−1^ can decrease grain yields; current recommendations suggest that salinity impacts most cultivated rice genotypes’ yield at or above 3.0 dSm^−1^ or around 30 mM NaCl [35,36]. For example, rice grown in soils with an ECe as low as 3.5 dSm^−1^ has been found to experience a yield loss of approximately 10%, and at an ECe of 7.2 dSm^−1^, yield loss can reach up to 50% [37]. According to the findings of another study, paddy rice yields begin to decrease at salinity levels of more than 3 dSm^−1^, after which a fall of 12% in yield may be anticipated for every 1 dS m1 increase in ECe [38]. Especially after the tsunami, a considerable geographical variation in the salinity level of ponded water was observed. The ECe in Japan ranges from 0.31 to 68.2 mScm^−1^, and it has been found that the salinity level varied greatly across the country [39]. In our study, a salt concentration of 75 mM was selected as the reference point for soil affected by a potential tsunami disaster, based on the salt concentration of the Japan Sea and Pacific Ocean [40] at VS (T_2_ and T_4_) and VS and RS (T_3_ and T_6_). Furthermore, in accordance with previous studies and our preliminary experiments, a single concentration of 75 mM NaCl was used as the salt stress in our investigation due to its close approximation to the LD_50_ value [37].

Based on the developmental phase, approximately 28 °C temperature is considered for the optimum growth and development of *Oryza sativa* L. [41,42]. The heat stress was applied at 31 °C, which refers to the global temperature rise [43]. The plants were exposed to heat stress only during the reproductive stage because of the most physiologically critical temperatures in the reproductive stage [44]. Additionally, multiple heat and salinity stresses were applied for the seen effect of multiple stress at RS in treatment 6 (T_6_) on rice genotypes.

Several abiotic stressors may have a cumulative or synergistic effect on plant development. Combined conditions, such as salinity and heat, are more damaging to plant growth than just one of these factors alone [19]. The first observation of the stress effect on plants is growth and photosynthesis performance under osmotic stress, which results from salinity and heat stress conditions [45,46,47]. While the rice plants can sense the stress, especially salinity stress, within five days at the seedling stage [31], bigger plants (at the vegetative stage) sense the salinity stress after three weeks in our experiment. After three weeks, salinity stress application devastates the plant growth of salt-sensitive genotypes (Yukinkomai and YNU SL). The Yukinkomai and YNU SL genotypes show different developmental responses under control conditions, and these differences can be welded to genotypic and environmental factors. Genetic copies of the plant genotype can exhibit strikingly distinct phenotypes under divergent naturalistic greenhouse conditions [48]. After eight weeks, the grain-filling stage and stress tolerance appear in the YNU31-2-4 genotype (Figure 1). Surprisingly, the heat stress at RS does not make any significant differences in plant growth performance because the plant growth was already completed at the reproductive stage (around 80 days old) to produce generative organs [49]. 

Photosynthesis is the most fundamental and intricate physiological event that directly affects plant growth and can be negatively affected by stressful environments such as heat and salinity [50]. The net photosynthesis rate (A_n_) was negatively affected by stress conditions in sensitive genotype YNU SL but primarily significantly increased in the YNU31-2-4 genotype. The YNU31-2-4 genotype showed significantly higher A_n_ under T_3_ (salinity at VS and RS) and T_6_ (salinity at VS and heat and salinity at RS) conditions (Figure 2 and Table A1 and Table A2). These stressors limit photosynthetic rate due to stomatal or nonstomatal constraints caused by stress [51,52]. For instance, in most green plants, drought stress, even at its low severity, can impede stomatal conductance and leaf photosynthesis [53]. The modulation of leaf stomatal conductance (g_s_) is an essential phenomenon in plants because it is necessary for desiccation avoidance and plant growth [54,55]. Although stress applications significantly reduce g_s_ in all genotypes, the g_s_ is higher in the YNU31-2-4 genotype than in other tested genotypes under T_3_ and T_6_ conditions (Figure 2 and Table A1 and Table A2).

The effects of osmotic stress caused by salinity on photosynthetic apparatus and metabolism are to be expected. Large concentrations of harmful ions, such as Na^+^ and Cl^−^, are known to damage thylakoid membranes when they accumulate in chloroplasts during salt stress [56,57]. The chloroplast is the binding site for photosynthesis, in which light and dark reactions occur. Salt stress can break down chlorophyll (Chl), the effect ascribed to an increased level of the toxic cation, Na^+^ [58,59,60,61]. Under salinity and multiple heat and salinity stress conditions, the YNU31-2-4 genotype shows a higher Chl accumulation (Figure 2 and Table A3 and Table A4). Although salt stress reduces the Chl concentration, the reduction level depends on the plant salt tolerance. For example, it is well known that Chl concentration increases in salt-tolerant species while decreasing in salt-sensitive species in saline regimes [62,63,64]. As a result, Chl accumulation has been recommended as one of the potential biochemical indicators of salt tolerance in various crops, such as wheat and rice [31,64]. 

Stressful environments have been shown to limit plant growth [65,66] and reduce crop yields through gas exchange characteristics [67,68,69]. In our experiment, salinity, heat, and heat and salinity stressors significantly reduce all harvesting parameters, grain size, and quality. The YNU31-2-4 genotype is less negatively affected than Yukinkomai and YNU SL genotypes under stressful environments. Although salinity and multiple heat and salinity stress reduce harvesting parameters, the YNU31-2-4 genotype shows higher PH, PB, RB, PN, PL, FLA, GNPP, SN, TGW, and YPP traits compared with other tested genotypes (Figure 3 and Table A5). Additionally, grain size and quality are higher in the YNU31-2-4 genotype than in the Yukinkomai and YNU SL genotypes under stress conditions (Figure 4 and Table A6). The heat and salinity stress causes substantial yield losses, and yield parameters are the primary tolerance determinant in crops [70]. It is seen that the YNU31-2-4 genotype has higher salinity and multiple heat and salinity stress tolerance capacity with higher yield and grain quality performance. Previously salt- and heat-tolerant genotypes also show similar responses under single stresses [71,72,73,74,75,76,77,78]. YNU31-2-4 genotype has a tolerance to salinity and heat stress and the tolerant capacity to multiple heat and salinity stress. The reason behind the tolerance mechanism of the YNU31-2-4 genotype can be seen in increasing CAT activity, lower toxic Na^+^ ion accumulation, and higher K^+^ accumulation (Figure 5 and Figure 6, and Table A6 and Table A7). Previously, we found low Na^+^ and high K^+^ accumulation as similar patterns under salt stress in the YNU31-2-4 genotype [31,79,80]. Related to salinity tolerance, we also find higher glucose accumulation in dry seeds of the YNU31-2-4 genotype; however, salinity and multiple heat and salinity stress reduce glucose accumulation in all tested genotypes (Figure 7). Glucose is a crucial signaling molecule in the stress tolerance mechanism, and higher glucose increases plant growth, photosynthesis, and salinity tolerance in plants [81]. 

This study’s findings reveal the YNU31-2-4 genotype’s ability to tolerate salinity and heat, thereby corroborating the efficacy of the PCA and HCA methodology in assessing stress tolerance in rice genotypes. As depicted in Figure 8, the stress treatments are categorized into T_1_ and T_5_, T_2_ and T_4_, and T_3_ and T_6_ conditions. However, the YNU31-2-4 genotype is located differently than other tested genotypes near the control (T_1_) treatment under T_2_, T_3_, T_4_, and T_6_ conditions. The PCA results indicated that the YNU31-2-4 genotype exhibits a greater capacity for stress tolerance. Furthermore, the Euclidean distance analysis reveals a correlation between the stress treatments and the separation of rice germplasms into more resilient and less resilient stress tolerance groups (Figure 8A, right-top panel) as previously described in other crop species [82,83]. The Yukinkomai and YNU SL genotypes are sensitive to salinity and heat and salinity stresses. Still, the YNU31-2-4 genotype shows a higher tolerance under both stress conditions with higher photosynthesis, CAT activity, and lower Na^+^ ion accumulation. 

## 4. Materials and Methods

### 4.1. Plant Material and Experimental Design

In this study, we used ‘Yukinkomai’ [84] and ‘YNU sister line (SL)’ [79] as salt-sensitive and ‘YNU31-2-4’ [80] as salt-tolerant rice genotypes. The ‘YNU31-2-4’ genotype was made by adding the hitomebore salt tolerant 1 (*hst1*) gene from “Kaijin” in an exact way using a method called “single nucleotide polymorphism (SNP) marker-assisted selection” (MAS). The SNP that caused the hst1 mutant line to tolerate high salt was found to be in the third exon of the Os06g0183100 gene, which is thought to code for a B-type response regulator called *OsRR22*. The ‘Yukinkomai’ genotype is a wild-type form of the ‘YNU31-2-4’ genotype [80], and the ‘YNU sister line (SL)’ is a salt-sensitive 99% identical genotype with ‘YNU31-2-4’ [79].

The rice seeds that had been stripped of their husks underwent a process of surface sterilization and were subsequently washed with a 2% hypochlorite solution (Fujifilm Wako Pure Chemical Corporation in Osaka, Japan). This process lasted for a duration of 20 min, after which the seeds were rinsed three times with sterile distilled water for a minute each time to eliminate any residual surface sterilization agents. The seeds that underwent sterilization were positioned on agar plates with a 1% concentration. These plates were supplemented with half of the Murashige and Skoog (MS) medium and were maintained at a pH of 5.8. The incubation process was carried out at a temperature of 26 °C. Ten-day-old seedlings were then transplanted to a tray with rice nursery culture soil containing 0.5 g N, 0.9 g P, and 0.5 g K kg^−1^ with the growth conditions 26/23 °C Day/night, 13/11 h day/night cycle, 350 µmol m^−2^ s^−1^ light intensity, and 70% relative humidity for 20 days. Thirty-day-old seedlings were transplanted into a 2.5 L pot containing rice nursery culture soil in the Kariwa Village Advanced Agro-Biotechnological Research Center (KAAB), Kashiwasaki, controlled growth chamber Niigata, Japan. Plants were grown at 26/23 °C Day/night temperature for 10 days to adapt to the greenhouse conditions. Forty-day-old uniform looking rice seedlings were accepted as the beginning of the vegetative stage and were subject to 6 different (Control; 26/23 °C, 0 mM NaCl, Salinity; 26/23 °C, 75 mM NaCl, Heat; 31/28 °C, 0 mM NaCl, and Heat and Salt; 31/28 °C, 75 mM NaCl) stress at different (SS; seedling stage, VS; vegetative stage, and RS; reproductive stage) growth periods (Table 1). The pots with plants under 75 mM salinity conditions (T_2_ and T_4_) were transferred to the 0 mM non-saline conditions after the removal of the salinity by washing to the control (26/23 °C) chamber (T_2_) or heat (31/28 °C) chamber (T_4_). Furthermore, the pots with plants under 0 mM non-saline conditions (26/23 °C) at the vegetative stage were transferred to a heat (31/28 °C) chamber at the reproductive stage (T_5_).

### 4.2. Sampling, Phenotyping, and Harvesting Determination

The experimental treatments were organized in a design that was completely randomized. Measurements were started a week after salt-stress treatment, and weekly plant growth was recorded. All experiments were performed in biological 3–5 replicates. Relative water content (RWC) was calculated according to Sade et al., (2009) from flag leaf as the following formula: %RWC = (FW–DM)/(Turgid Weight–DM) × 100. 

The main agronomic traits such as plant height (PH), plant biomass (PB), root length (RL), root biomass (RB), panicle number (PN), panicle length (PL), flag leaf area (FLA), grain number per panicle (GNPP), spikelet number (SN), 1000-grain weight (TG), and yield per plant (YPP) were measured at the physiological maturity of grains. They were determined in 5 plants per genotype and treatment. Perfect grain (PG), chalky grain (CG), grain length (GL), grain width (GW), and grain thickness (GT) were determined as three replicates of 300-seed samples of each genotype for each treatment with a rice grain grader (RGQI20A, Satake, Hiroshima, Japan).

### 4.3. Chlorophyll Content and Leaf Gas Exchange Measurements

Chlorophyll pigments chlorophyll a (Chla), chlorophyll b (Chl b), and total chlorophyll (ChlT) contents were determined using the method of Hori et al. [85].

A portable photosynthesis LI-6400XL equipment was used to assess leaf gas exchange (LI-6400-20, LiCor Biosciences, Lincoln, NE, USA). The net photosynthetic rate (A_n_) (μmol CO_2_ m^−2^ s^−1^), stomatal conductance (g_s_) (mmol m^−2^ s^−1^), transpiration rate (E) (mmol m^−2^ s^−1^), intercellular CO_2_ concentration (C_i_), and the ratio of intercellular to ambient CO_2_ concentration (C_i_/C_a_) of the flag leaves (fully expanded functional leaves) were measured when the active photosynthetic radiation (PAR) was ≥1000 µmol m^−2^ s^−1^ to ensure maximum values at sunny without cloudy days during 9:00–13:00 and relative humidity ranging between 45–55%, a leaf temperature of 26 °C (NT) and 31 °C (HT) at the flowering stage. The ratio of A_n_/E, which is the quantity of CO_2_ fixed per unit amount of water lost by transpiration, was used to compute the instantaneous water usage efficiency (WUE). Leaf gas exchange measurements were taken from five flag leaves of rice plants for each treatment.

### 4.4. Malondialdehyde (MDA), Proline, Protein, and Antioxidant Enzyme Activities

The free proline content was determined using a modified version of the method described by Bates et al. [86]. Briefly, 0.5 g of fresh leaf samples were homogenized in 10 mL of 3% sulfosalicylic acid and incubated at 4 °C for 24 h. The homogenate was centrifuged at 10,000× *g* at 25 °C for 5 min, and the supernatant (1 mL) was reacted with 1 mL of ninhydrin reagent and 1 mL of glacial acetic acid in a test tube at 100 °C for 1 h. The reaction was interrupted by placing the test tubes in an ice bath for 20 min. The proline was extracted with 2 mL of toluene and incubated for 30 min at ambient temperature. The toluene phase was discarded, and the absorbance at 520 nm was measured with a double beam spectrophotometer U-2900. (Hitachi, Tokyo, Japan).

The quantification of Malondialdehyde (MDA) was conducted using a refined technique developed by Dhindsa and Matowe [87]. In summary, the leaf specimen weighing 0.5 g was subjected to homogenization using 5 mL of 0.1% trichloroacetic acid and subsequently centrifuged at 12,500× *g* at a temperature of 25 °C for a duration of 20 min. Two milliliters of supernatant were combined with two milliliters of thiobarbituric acid-TCA. The mixture underwent incubation at a temperature of 90 °C for a duration of 30 min, following which the reaction was terminated by transferring the tube to an ice bath for a period of 10 min. The chromogen was quantified at wavelengths of 520 and 600 nm utilizing a double beam spectrophotometer model U-2900. (Hitachi, Tokyo, Japan).

The frozen leaf powder samples weighing 1 g were subjected to homogenization in a cold mortar using 4 milliliters of 1 molar phosphate buffer with a pH of 7.0. The buffer contained 0.1 millimolar of Na-EDTA. (10 mL). The homogenate underwent centrifugation at a force of 15,000 times the acceleration due to gravity for a duration of 15 min at a temperature of 4 degrees Celsius. The resulting supernatant was utilized for the purpose of quantifying antioxidant enzyme activities, as described in reference [88]

The protein concentration was determined by using a Bradford Protein Assay Kit (Bio-Rad Laboratories GmbH, Hercules, CA, USA).

The determination of Catalase (CAT) activity was carried out by observing the rate of reduction in absorbance at 240 nm over a period of three minutes subsequent to the utilization of H_2_O_2_ [89]. The experimental setup involved combining 0.8 mL of a 50 mM phosphate buffer solution (pH 7.6) containing 0.1 mM Na-EDTA, 0.1 mL of 100 mM H_2_O_2_, and 0.1 mL of enzyme extract in a 2 mL volume to form the reaction mixture.

The determination of the Superoxide Dismutase (SOD) method employed by Cakmak and Marschner [90] is carried out by measuring its ability to inhibit the photochemical reduction of nitro blue tetrazolium (NBT). The definition of a single unit of SOD was established as the quantity of the enzyme necessary to elicit a 50% reduction in NBT reduction at a temperature of 25 °C. The expression of Superoxide Dismutase (SOD) activity was quantified in units per minute per gram of fresh weight (FW). The measurement of absorbance was conducted at a wavelength of 650 nm utilizing a double beam spectrophotometer model U-2900. (Hitachi, Tokyo, Japan).

The activity of ascorbate peroxidase (APX) was evaluated by measuring the reduction in absorbance at 290 nm over a period of 1 min, using the method described by Amako et al. [91]. The experimental solution comprised 100 μL of extract sample, 50 mM potassium phosphate buffer (pH 7.6), 0.5 mM H_2_O_2_, and 0.1 mM ascorbate. The enzymatic reaction was commenced through the introduction of the enzyme extract, and subsequently, the reduction in absorbance was documented.

### 4.5. Na^+^ and K^+^ Measurement

The quantification of sodium (Na^+^) and potassium (K^+^) ions in shoots and roots was performed using a wet digestion method [92]. Plant samples, which were dried and finely powdered, weighing 10 mg, underwent digestion in a solution of HNO_3_. The specimens were subjected to incubation in a thermal bath at a temperature of 60 °C for a duration of 2 h. Following the cooling process, hydrogen peroxide was introduced to the digestion solution and subsequently subjected to heating within the range of 60 to 120 °C. The solution that underwent digestion was subjected to gentle shaking and filtration using 0.2-µm filters (Whatman, Maidstone, UK), with the solid residue being excluded. The quantification of Na^+^ and K^+^ contents in the extract was performed using Polarized Zeeman Atomic Absorption spectrophotometry. (Z-6100, Hitachi, Tokyo, Japan).

### 4.6. Measurement of Glucose Content

The measurement of glucose was done using the modified protocol of Kaneko et al. [93]. In brief, 50 mg of rice flour sample was taken into a 2 mL tube and treated with 0.5 mL of 80% ethanol and boiling dry heat bath for 5 min, and the mixture was centrifuged at 12,000× *g* for 10 min. The supernatant was boiled again for 20 min. The ethanol extraction process was repeated two times. The boiled supernatant was wholly dried in a vacuum concentrator, and then 60 µL ultrapure water was added to measure the free sugar extraction. The soluble glycan was hydrolyzed by 5 units of amyloid glycosidase and 1 unit of α-amylase, and the released glucose from soluble glycan was measured by a coupled enzyme reaction using hexokinase (HK) and Glc-6-P dehydrogenase (G6PDH) [94]. The assay mixture, composed of 100 mM Tris-HCl (pH 7.6), 3 mM MgCl_2_, 2 mM ATP, 0.6 mM NAD^+^, 1 unit of HK, and 1 unit of G6PDH, was incubated at 37 °C for 30 min. After chilling, a spectrophotometer was used to detect absorbance at 340 nm (NanoDrop One^C^, Thermo Fisher Scientific, Waltham, MA, USA).

### 4.7. Statistical Analysis

The recorded data average for each trait is standardized, obtaining a ratio (Treatment/Control). To evaluate differences between genotypes and environments (treatments), collected data are submitted to a two-way analysis of variance (ANOVA) using R software (V3.6.1, https://www.r-project.org/, accessed on 8 March 2023). Tukey’s honest significant difference (HSD) test at *p* < 0.05 is used with R software, including the ‘glht’ function in the ‘multcomp’ package [95]. The correlation matrix of 3 genotypes and 6 different treatments is subjected to principal component analysis (PCA). The index values for each treatment are first determined by comparing the stress reaction to the control value. For the PCA analysis, all of the traits in each treatment re merged and used as index values. These index values are used to determine the ordination space association of response variable vectors and genotypes. A two-way heatmap clustering analysis (HCA) is performed on the same dataset with PCA analysis. To compute the dissimilarity matrix, Pearson correlation and a ‘euclidean algorithm’ are used. PCA and HCA re generated using the R software, specifically the ‘prcomp’ function in the ‘factoextra’ library [96]. The heatmap function in the ‘pheatmap’ library with R software is used to organize data hierarchically [97]. 

## 5. Conclusions

All stress scenarios applied at vegetative and reproductive stages reduce the plant growth performance and yield in all genotypes. Interestingly, heat stress during the reproductive stage has little effect on rice genotype yield performance but does impair grain quality indicators. While salt and repeated heat and salinity stress have a substantial impact on yield performance and grain quality in the Yukinkomai and YNU SL genotypes, the YNU31-2-4 genotype demonstrates a greater yield and grain quality under heavy stress. The data show that the YNU31-2-4 genotype accumulates less Na^+^ and more K^+^ under salinity and various stresses. In the YNU31-2-4 genotype, low-level harmful ion accumulation leads to increased photosynthetic activity and pigment accumulation, which promotes yield capacity. Likewise, whereas stress reduces glucose accumulation in dry seeds, the YNU31-2-4 genotype shows an increased glucose accumulation in dry seeds.

## Figures and Tables

**Table 1:** A diagram illustrating of stress on rice crops at different stages of growth.

Treatment (T)	Seedling Stage (SS)	Vegetative Stage (VS)	Reproductive Stage (RS)
T_1_	Control (C; 26 ℃, 0 mM NaCl)	Control (C; 26 °C, 0 mM NaCl)	Control (C; 26 ℃, 0 mM NaCl)
T_2_	Control (C; 26 ℃, 0 mM NaCl)	Salinity (S; 26 ℃, 75 mM NaCl)	Control (C; 26 ℃, 0 mM NaCl)
T_3_	Control (C; 26 ℃, 0 mM NaCl)	Salinity (S; 26 ℃, 75 mM NaCl)	Salinity (S; 26 ℃, 75 mM NaCl)
T_4_	Control (C; 26 ℃, 0 mM NaCl)	Salinity (S; 26 ℃, 75 mM NaCl)	Heat (H; 31 ℃, 0 mM NaCl)
T_5_	Control (C; 26 ℃, 0 mM NaCl)	Control (C; 26 ℃, 0 mM NaCl)	Heat (H; 31 ℃, 0 mM NaCl)
T_6_	Control (C; 26 ℃, 0 mM NaCl)	Salinity (S; 26 ℃, 75 mM NaCl)	Heat and Salinity (H + S; 31 ℃, 75 mM NaCl)

## Data Availability

Not applicable.

## Supplementary Materials

The following supporting information can be downloaded at: https://www.mdpi.com/article/10.3390/plants12091910/s1, Table S1: Variance analysis tables; Table S2: Loading values and percentage contribution of variables on the axis identified by the principal component analysis (PCA) for all cultivars under control and saline conditions. Figure S1: The plant and tiller images of genotypes under (A) T_1_, (B) T_2_, (C) T_3_, (D) T_4_, (E) T_5_, and (F) T_6_ conditions.

## Appendix A

**Table A1:** Photosynthetic parameters (the net photosynthetic rate, An; stomatal conductance, g_s_; the intercellular CO_2_ concentration, C_i_; transpiration rate, E; and the ratio of intercellular to ambient CO_2_ concentration, C_i_/C_a_) of Yukinkomai, YNU31-2-4, and YNU SL rice genotypes under treatment 1 (T_1_), T_2_, T_3_, T_4_, T_5_, and T_6_. The Tukey HSD test from three independent biological replicates (*n* = 3) shows that means (±SD) in the same graph followed by letters are substantially different at *p* < 0.05.

		A_n_	g_s_	C_i_	E	C_i_/C_a_
T_1_	Yukinkomai	27.24 ± 2.143 b	0.262 ± 0.008 ce	195.094 ± 15.722 ce	0.005 ± 0.001 cd	0.502 ± 0.038 fg
	YNU31-2-4	36.565 ± 0.735 df	0.294 ± 0.014 de	270.645 ± 16.142 i	0.006 ± 0.001 d	0.646 ± 0.024 hi
	YNU SL	51.712 ± 4.159 hi	0.447 ± 0.016 g	186.313 ± 17.761 cd	0.008 ± 0.000 fg	0.762 ± 0.021 j
T_2_	Yukinkomai	47.030 ± 0.903 gh	0.444 ± 0.040 g	258.815 ± 32.877 hi	0.006 ± 0.001 def	0.692 ± 0.051 ij
	YNU31-2-4	35.554 ± 3.414 bde	0.341 ± 0.027 ef	198.411 ± 9.735 ce	0.006 ± 0.001 def	0.275 ± 0.043 cd
	YNU SL	59.379 ± 3.999 j	0.651 ± 0.033 i	213.957 ± 10.706 deg	0.006 ± 0.001 def	0.563 ± 0.029 gh
T_3_	Yukinkomai	39.280 ± 2.290 ef	0.251 ± 0.024 cd	164.123 ± 9.049 c	0.003 ± 0.000 ac	0.404 ± 0.039 ef
	YNU31-2-4	48.602 ± 2.046 gh	0.235 ± 0.017 cd	69.973 ± 7.970 ab	0.005 ± 0.001 bcd	0.190 ± 0.014 bc
	YNU SL	17.033 ± 1.216 a	0.073 ± 0.020 a	93.351 ± 3.303 b	0.002 ± 0.000 a	0.266 ± 0.027 cd
T_4_	Yukinkomai	57.288 ± 1.646 ij	0.462 ± 0.026 g	166.029 ± 16.803 c	0.008 ± 0.001 eg	0.502 ± 0.074 fg
	YNU31-2-4	34.367 ± 5.005 cde	0.212 ± 0.027 bc	247.628 ± 14.765 gi	0.005 ± 0.001 cd	0.214 ± 0.055 bd
	YNU SL	47.027 ± 2.426 gh	0.258 ± 0.026 cd	199.569 ± 17.701 cef	0.006 ± 0.001 de	0.317 ± 0.039 de
T_5_	Yukinkomai	33.770 ± 1.422 bde	0.420 ± 0.011 fg	245.587 ± 1.999 gi	0.011 ± 0.000 h	0.633 ± 0.007 hi
	YNU31-2-4	43.255 ± 1.740 fg	0.468 ± 0.018 fh	228.309 ± 3.413 egh	0.010 ± 0.001 gh	0.598 ± 0.010 gi
	YNU SL	38.179 ± 1.264 df	0.546 ± 0.062 h	239.284 ± 2.934 fgi	0.010 ± 0.000 h	0.681 ± 0.020 ij
T_6_	Yukinkomai	28.802 ± 0.807 bc	0.115 ± 0.018 a	30.912 ± 2.471 a	0.004 ± 0.000 ac	0.080 ± 0.005 a
	YNU31-2-4	52.535 ± 1.267 hj	0.233 ± 0.029 cd	53.508 ± 2.453 ab	0.005 ± 0.000 cd	0.131 ± 0.010 ab
	YNU SL	31.766 ± 0.875 bd	0.144 ± 0.009 ab	35.912 ± 1.767 a	0.003 ± 0.000 ab	0.074 ± 0.009 a

**Table A2:** Photosynthetic parameters (Water use efficiency, WUE (A_n_/g_s_); chlorophyll a, Chla; chlorophyll b, Chlb; total chlorophyll, chlT content; and relative water content, RWC) of Yukinkomai, YNU31-2-4, and YNU SL rice genotypes under treatment 1 (T_1_), T_2_, T_3_, T_4_, T_5_, and T_6_. The Tukey HSD test from three independent biological replicates (*n* = 3) shows that means (±SD) in the same graph followed by letters are substantially different at *p* < 0.05.

		WUE	Chla	Chlb	ChlT	RWC
T_1_	Yukinkomai	5356.450 ± 222.552 acd	173.194 ± 0.716 e	153.654 ± 3.269 fgh	151.35 ± 2.741 fghi	82.158 ± 4.546 bcd
	YNU31-2-4	6420.039 ± 658.935 bce	168.620 ± 3.417 e	214.242 ± 22.012 ik	199.261 ± 17.123 ijl	86.986 ± 2.844 d
	YNU SL	6525.314 ± 363.796 bce	164.792 ± 4.350 de	236.165 ± 21.879 jk	216.246 ± 16.866 kl	81.667 ± 2.357 cd
T_2_	Yukinkomai	7405.811 ± 1249.448 deg	152.335 ± 6.312 de	93.577 ± 5.494 bcde	101.040 ± 4.813 cde	83.397 ± 5.656 cd
	YNU31-2-4	5862.909 ± 808.315 ade	174.102 ± 1.434 e	148.976 ± 12.820 eg	147.740 ± 10.525 egh	87.774 ± 3.754 d
	YNU SL	9635.331 ± 1925.732 fgi	157.486 ± 6.956 de	131.329 ± 33.090 dg	136.017 ± 32.179 dg	75.797 ± 9.239 bcd
T_3_	Yukinkomai	11,742.127 ± 1884.126 i	104.073 ± 1.979 b	65.102 ± 15.081 ac	69.014 ± 15.757 ac	68.004 ± 7.417 ac
	YNU31-2-4	10,252.976 ± 1707.586 gi	154.695 ± 7.976 de	105.15 ± 3.273 cdef	109.415 ± 3.497 cdef	77.548 ± 2.260 bcd
	YNU SL	9511.533 ± 314.831 fgi	59.195 ± 16.011 a	33.682 ± 12.376 a	35.349 ± 13.308 a	65.027 ± 7.742 ab
T_4_	Yukinkomai	7455.155 ± 686.156 deg	141.545 ± 4.980 cd	86.637 ± 0.722 ad	92.099 ± 0.676 bcd	73.158 ± 6.835 ad
	YNU31-2-4	6860.766 ± 627.490 cef	169.760 ± 7.829 e	217.640 ± 37.459 ik	201.802 ± 28.990 jl	79.069 ± 4.280 bcd
	YNU SL	8246.898 ± 1111.925 egh	151.440 ± 14.435 ce	119.849 ± 10.642 cdg	123.383 ± 12.547 dg	78.278 ± 2.683 bcd
T_5_	Yukinkomai	3198.187 ± 24.146 a	163.181 ± 8.136 de	265.235 ± 28.815 k	242.345 ± 25.235 l	88.549 ± 2.595 d
	YNU31-2-4	4514.127 ± 518.664 ac	163.873 ± 2.306 de	206.358 ± 13.852 hij	189.627 ± 14.032 hjk	86.706 ± 2.406 d
	YNU SL	3646.112 ± 265.588 ab	172.111 ± 0.592 e	173.085 ± 9.185 gi	166.777 ± 7.280 gj	84.553 ± 2.596 cd
T_6_	Yukinkomai	8192.701 ± 337.352 degh	128.363 ± 12.348	84.637 ± 17.380 ad	87.566 ± 14.384 bcd	72.563 ± 1.532 ad
	YNU31-2-4	9935.842 ± 518.927 gi	163.280 ± 2.355	168.113 ± 27.65 gi	161.552 ± 22.384 gj	76.905 ± 8.693 bcd
	YNU SL	10,534.637 ± 282.673 hi	78.732 ± 7.221	39.708 ± 4.669 ab	44.599 ± 4.878 ab	57.663 ± 11.171 a

**Table A3:** Harvesting parameters (plant height, plant biomass, root length, root biomass, panicle number, panicle length, flag leaf area, grain number per panicle, spikelet number, 1000-grain weight, and yield per plant) of Yukinkomai, YNU31-2-4, and YNU SL rice genotypes under treatment 1 (T_1_), T_2_, T_3_, T_4_, T_5_, and T_6_. The Tukey HSD test from three independent biological replicates (*n* = 3) shows that means (±SD) in the same graph followed by letters are substantially different at *p* < 0.05.

		Plant Height	Plant Biomass	Root Length	Root Biomass	Panicle Number	Panicle Length
T_1_	Yukinkomai	116.120 ± 4.348 b	125.366 ± 4.282 k	34.440 ± 1.766 h	17.792 ± 2.179 eg	22.400 ± 1.517 f	17.791 ± 0.335 h
	YNU31-2-4	125.480 ± 2.777 c	112.752 ± 3.730 j	34.800 ± 1.483 hi	19.830 ± 1.598 fg	21.800 ± 0.837 f	18.313 ± 0.591 hi
	YNU SL	120.540 ± 3.957 bc	104.315 ± 2.263 ij	28.200 ± 2.168 df	16.127 ± 2.948 def	21.000 ± 1.581 f	18.929 ± 0.634 i
T_2_	Yukinkomai	89.620 ± 2.309 a	35.323 ± 2.772 ce	25.440 ± 1.901 ade	3.774 ± 0.522 a	19.200 ± 1.643 ef	12.565 ± 0.237 de
	YNU31-2-4	113.480 ± 3.989 b	98.184 ± 3.310 hi	33.970 ± 2.815 gh	13.127 ± 2.666 cd	27.400 ± 3.209 g	15.281 ± 0.238 fg
	YNU SL	92.740 ± 3.686 a	38.495 ± 3.370 de	25.000 ± 2.000 ade	4.004 ± 0.641 a	20.600 ± 2.793 f	13.500 ± 0.141 e
T_3_	Yukinkomai	91.340 ± 1.685 a	21.620 ± 2.534 ab	22.900 ± 1.949 abc	4.520 ± 0.820 a	7.000 ± 1.732 ab	10.953 ± 0.345 ab
	YNU31-2-4	116.680 ± 4.218 bc	53.845 ± 4.672 f	25.400 ± 1.380 ade	9.350 ± 2.448 bc	14.800 ± 2.280 de	14.639 ± 0.232 f
	YNU SL	92.320 ± 4.322 a	19.351 ± 4.236 ab	21.200 ± 2.308 a	3.359 ± 1.300 a	5.600 ± 2.702 ab	11.282 ± 0.824 ac
T_4_	Yukinkomai	95.400 ± 1.319 a	37.731 ± 6.384 de	27.420 ± 1.675 cdf	3.856 ± 0.137 a	18.400 ± 1.140 ef	12.307 ± 0.358 cd
	YNU31-2-4	117.760 ± 4.091 bc	79.920 ± 4.778 g	31.600 ± 2.074 fh	10.491 ± 1.124 c	22.400 ± 1.342 f	15.827 ± 0.685 g
	YNU SL	89.640 ± 1.713 a	33.379 ± 4.449 cd	26.000 ± 1.837 bde	2.584 ± 0.975 a	21.200 ± 1.789 f	11.965 ± 0.272 bcd
T_5_	Yukinkomai	118.140 ± 1.435 bc	93.131 ± 3.345 h	24.000 ± 1.581 ad	14.375 ± 2.385 de	18.600 ± 1.673 ef	18.721 ± 0.137 hi
	YNU31-2-4	122.300 ± 2.432 bc	106.441 ± 5.899 ij	39.200 ± 2.588 i	20.162 ± 1.942 g	18.400 ± 0.894 ef	18.595 ± 0.165 hi
	YNU SL	117.720 ± 8.164 bc	103.708 ± 3.477 ij	29.600 ± 2.881 efg	12.959 ± 1.989 cd	21.800 ± 1.304 f	18.517 ± 0.287 hi
T_6_	Yukinkomai	95.120 ± 8.007 a	25.953 ± 2.461 bc	20.800 ± 2.864 a	3.683 ± 1.056 a	10.000 ± 3.937 bc	12.018 ± 0.602 bcd
	YNU31-2-4	120.340 ± 2.791 bc	44.673 ± 6.520 ef	26.440 ± 1.170 bde	6.147 ± 1.218 ab	11.800 ± 2.588 cd	15.825 ± 0.812 g
	YNU SL	95.180 ± 2.194 a	13.672 ± 4.876 a	22.000 ± 1.581 ab	3.223 ± 0.703 a	3.200 ± 1.643 a	10.716 ± 0.612 a

**Table A4:** Harvesting parameters (flag leaf area, grain number per panicle, spikelet number, 1000-grain weight, and yield per plant) of Yukinkomai, YNU31-2-4, and YNU SL rice genotypes under treatment 1 (T_1_), T_2_, T_3_, T_4_, T_5_, and T_6_. The Tukey HSD test from three independent biological replicates (*n* = 3) shows that means (±SD) in the same graph followed by letters are substantially different at *p* < 0.05.

		Flag Leaf Area	Grain Number per Panicle	Spikelet Number	1000-Grain Weight	Yield per Plant
T_1_	Yukinkomai	48.637 ± 2.186 bd	75.260 ± 5.974 gh	76.894 ± 6.279 e	26.677 ± 0.911 jk	40.502 ± 3.234 gh
	YNU31-2-4	52.717 ± 2.521 d	66.495 ± 3.762 g	71.588 ± 4.566 e	28.055 ± 0.298 k	36.941 ± 1.740 g
	YNU SL	46.600 ± 2.623 bd	80.511 ± 6.279 h	82.908 ± 5.714 e	25.673 ± 0.645 ij	40.822 ± 1.943 gh
T_2_	Yukinkomai	25.153 ± 3.798 a	13.523 ± 2.564 ac	30.755 ± 3.580 ab	22.340 ± 0.352 efg	6.069 ± 0.678 cd
	YNU31-2-4	45.527 ± 2.781 bd	41.892 ± 5.801 f	57.665 ± 7.070 d	26.186 ± 0.242 ik	28.347 ± 4.024 f
	YNU SL	19.183 ± 1.207 a	11.775 ± 4.717 ab	36.068 ± 7.582 ab	20.837 ± 0.577 cdf	9.211 ± 1.930 d
T_3_	Yukinkomai	21.560 ± 3.175 a	3.677 ± 2.505 a	39.364 ± 6.884 abc	18.918 ± 0.671 bc	0.430 ± 0.288 ab
	YNU31-2-4	39.233 ± 1.405 b	30.127 ± 7.227 de	50.889 ± 6.692 cd	23.421 ± 0.242 gh	10.024 ± 1.773 d
	YNU SL	22.720 ± 4.095 a	7.447 ± 3.387 a	40.917 ± 7.252 abc	16.083 ± 0.520 a	0.606 ± 0.265 ab
T_4_	Yukinkomai	19.520 ± 6.323 a	19.751 ± 4.089 bcd	28.338 ± 7.168 a	20.399 ± 0.401 bde	7.189 ± 1.643 d
	YNU31-2-4	39.973 ± 3.470 bc	32.939 ± 7.145 ef	44.211 ± 8.162 bd	27.615 ± 0.511 jk	20.247 ± 4.246 e
	YNU SL	26.173 ± 2.317 a	13.260 ± 3.501 ac	27.477 ± 1.645 a	21.715 ± 0.537 dfg	5.619 ± 1.221 bcd
T_5_	Yukinkomai	52.857 ± 3.170 d	72.552 ± 4.786 gh	74.229 ± 4.866 e	25.744 ± 0.671 ij	41.552 ± 4.508 gh
	YNU31-2-4	49.530 ± 1.924 cd	74.066 ± 2.634 gh	80.502 ± 3.817 e	27.760 ± 0.147 k	37.811 ± 1.754 gh
	YNU SL	51.910 ± 4.556 d	80.670 ± 2.711 h	83.154 ± 2.068 e	24.467 ± 0.298 hi	43.067 ± 1.837 h
T_6_	Yukinkomai	19.093 ± 4.575 a	5.503 ± 4.605 a	37.059 ± 6.249 ab	19.809 ± 0.632 bd	1.135 ± 0.787 ac
	YNU31-2-4	45.493 ± 3.169 bd	23.268 ± 8.025 ce	39.022 ± 3.049 abc	22.557 ± 1.242 fh	6.386 ± 2.514 cd
	YNU SL	23.233 ± 0.907 a	4.300 ± 2.273 a	29.030 ± 7.712 a	18.475 ± 1.305 b	0.230 ± 0.200 a

**Table A5:** Grain quality parameters (Grain length, grain width, grain thickness, perfect grain, and chalky grain) of Yukinkomai, YNU31-2-4, and YNU SL rice genotypes under treatment 1 (T_1_), T_2_, T_3_, T_4_, T_5_, and T_6_. The Tukey HSD test from three independent biological replicates (*n* = 3) shows that means (±SD) in the same graph followed by letters are substantially different at *p* < 0.05.

		Perfect Grain	Chalky Grain	Grain Length	Grain Width	Grain Thickness
T_1_	Yukinkomai	62.350 ± 0.071 f	34.533 ± 0.321 a	5.127 ± 0.016 h	2.737 ± 0.006 g	2.007 ± 0.006 ghi
	YNU31-2-4	16.567 ± 3.353 bd	83.100 ± 3.504 fhj	5.080 ± 0.010 h	2.853 ± 0.006 hi	2.057 ± 0.015 ij
	YNU SL	53.433 ± 3.355 f	44.167 ± 3.528 ab	5.073 ± 0.006 h	2.770 ± 0.010 gh	1.967 ± 0.058 eh
T_2_	Yukinkomai	19.900 ± 1.637 cd	58.667 ± 5.033 bcd	4.750 ± 0.020 de	2.440 ± 0.017 d	1.880 ± 0.000 bcd
	YNU31-2-4	24.133 ± 1.159 de	72.667 ± 3.512 deh	5.100 ± 0.000 h	2.867 ± 0.006 hi	2.117 ± 0.006 jk
	YNU SL	32.867 ± 5.129 e	97.333 ± 15.144 j	4.873 ± 0.012 eg	2.567 ± 0.021 e	1.933 ± 0.006 def
T_3_	Yukinkomai	5.433 ± 2.892 a	66.600 ± 5.717 cefg	4.510 ± 0.095 ac	2.300 ± 0.026 ab	1.760 ± 0.035 a
	YNU31-2-4	12.467 ± 1.686 abc	83.667 ± 1.650 ghj	4.897 ± 0.021 fg	2.710 ± 0.000 fg	1.983 ± 0.006 fh
	YNU SL	8.000 ± 10.046 ab	63.933 ± 8.701 ce	4.420 ± 0.140 a	2.327 ± 0.090 bc	1.830 ± 0.017 ab
T_4_	Yukinkomai	4.167 ± 0.321 a	85.233 ± 1.595 hj	4.603 ± 0.006 bc	2.433 ± 0.012 cd	1.957 ± 0.006 efg
	YNU31-2-4	8.500 ± 1.609 ab	89.000 ± 1.179 hj	5.067 ± 0.006 h	2.883 ± 0.006 i	2.140 ± 0.010 k
	YNU SL	7.867 ± 0.351 ab	65.567 ± 1.79 cef	4.547 ± 0.015 ac	2.333 ± 0.021 bd	1.900 ± 0.010 be
T_5_	Yukinkomai	35.033 ± 1.401 e	63.000 ± 0.721 ce	5.133 ± 0.006 h	2.713 ± 0.015 fg	2.057 ± 0.006 ij
	YNU31-2-4	7.433 ± 2.228 ab	91.267 ± 2.228 ij	5.103 ± 0.012 h	2.850 ± 0.000 hi	2.143 ± 0.006 k
	YNU SL	6.767 ± 0.850 ab	90.967 ± 0.950 ij	5.000 ± 0.000 gh	2.623 ± 0.015 ef	2.037 ± 0.006 hi
T_6_	Yukinkomai	3.000 ± 1.000 a	77.133 ± 0.902 ehi	4.637 ± 0.021 cd	2.330 ± 0.030 bc	1.920 ± 0.017 cef
	YNU31-2-4	9.933 ± 0.503 abc	80.567 ± 1.007 ehj	4.850 ± 0.017 ef	2.557 ± 0.012 e	2.020 ± 0.010 ghi
	YNU SL	4.167 ± 7.217 a	53.633 ± 13.359 bc	4.470 ± 0.092 ab	2.200 ± 0.104 a	1.857 ± 0.060 bc

**Table A6:** Protein, malondialdehyde (MDA), proline (PRO) content, catalase (CAT), superoxide dismutase (SOD), and ascorbate peroxidase (APX) activity of Yukinkomai, YNU31-2-4, and YNU SL rice genotypes under treatment 1 (T_1_), T_2_, T_3_, T_4_, T_5_, and T_6_. The Tukey HSD test from three independent biological replicates (*n* = 3) shows that means (±SD) in the same graph followed by letters are substantially different at *p* < 0.05.

		Protein	MDA	PRO	CAT	SOD	APX
T_1_	Yukinkomai	3050.106 ± 68.337 bd	6.237 ± 0.453 a	0.533 ± 0.024 ad	2.401 ± 0.337 ab	23.676 ± 1.074 ef	157.972 ± 6.912 bc
	YNU31-2-4	2938.287 ± 78.909 abc	9.281 ± 0.901 bd	0.409 ± 0.055 a	2.045 ± 0.013 a	10.386 ± 0.913 abc	254.73 ± 14.490 d
	YNU SL	3361.854 ± 39.895 ef	11.452 ± 1.137 df	0.739 ± 0.034 bd	15.632 ± 1.557 e	7.023 ± 0.267 a	169.462 ± 20.805 bc
T_2_	Yukinkomai	2782.591 ± 65.920 ab	9.274 ± 0.980 bd	0.859 ± 0.066 d	2.671 ± 0.430 ab	8.539 ± 0.103 a	145.742 ± 7.114 bc
	YNU31-2-4	3178.910 ± 142.256 cdf	11.741 ± 0.979 df	0.385 ± 0.039 a	31.873 ± 5.326 h	8.106 ± 0.773 a	79.500 ± 11.281 a
	YNU SL	2868.295 ± 74.170 ab	13.467 ± 1.310 f	0.495 ± 0.018 abc	20.659 ± 1.769 fg	11.198 ± 0.645 abc	125.077 ± 10.497 ab
T_3_	Yukinkomai	2703.326 ± 68.249 a	12.519 ± 0.943 ef	3.401 ± 0.301 g	7.077 ± 0.516 bd	67.569 ± 1.149 h	354.956 ± 48.535 ef
	YNU31-2-4	3144.940 ± 49.216 cde	11.607 ± 0.862 df	2.411 ± 0.094 f	21.514 ± 1.351 g	10.682 ± 0.676 abc	125.488 ± 11.578 ab
	YNU SL	2833.404 ± 124.041 ab	9.667 ± 0.757 bd	0.787 ± 0.047 cd	18.677 ± 0.735 eg	17.053 ± 1.275 cde	147.717 ± 5.814 bc
T_4_	Yukinkomai	2853.503 ± 74.106 ab	7.393 ± 1.054 ab	0.430 ± 0.060 ab	16.007 ± 0.795 ef	9.761 ± 1.454 ab	176.568 ± 25.973 bc
	YNU31-2-4	3224.983 ± 84.522 df	9.571 ± 0.334 bd	0.446 ± 0.023 ab	10.385 ± 0.903 d	8.358 ± 1.377 a	251.031 ± 21.805 d
	YNU SL	2844.215 ± 55.318 ab	7.504 ± 0.862 ab	0.809 ± 0.034 cd	5.658 ± 0.450 abc	5.236 ± 0.495 a	204.629 ± 8.371 cd
T_5_	Yukinkomai	3363.057 ± 78.570 ef	11.763 ± 0.312 df	0.446 ± 0.033 ab	5.408 ± 0.216 abc	27.371 ± 0.74 f	475.974 ± 18.797 gh
	YNU31-2-4	3238.358 ± 50.660 df	9.178 ± 0.640 bd	0.378 ± 0.032 a	5.697 ± 0.895 ad	40.458 ± 7.777 g	527.775 ± 16.803 h
	YNU SL	3650.955 ± 191.165 g	10.659 ± 0.634 de	0.497 ± 0.073 abc	18.724 ± 1.190 eg	22.567 ± 1.508 df	134.957 ± 4.541 ab
T_6_	Yukinkomai	2683.227 ± 58.142 a	10.200 ± 0.954 cde	0.795 ± 0.090 cd	8.535 ± 1.023 cd	21.685 ± 1.503 df	322.044 ± 24.748 e
	YNU31-2-4	3431.423 ± 94.090 fg	10.000 ± 0.794 bde	0.667 ± 0.060 ad	6.048 ± 0.376 ad	15.865 ± 1.491 bd	418.579 ± 38.202 fg
	YNU SL	2762.774 ± 37.099 a	7.882 ± 1.148 abc	1.664 ± 0.264 e	4.710 ± 0.679 abc	25.791 ± 3.874 f	457.374 ± 20.386 g

**Table A7:** Ion content (root and shoot) of Yukinkomai, YNU31-2-4, and YNU SL rice genotypes under treatment 1 (T_1_), T_2_, T_3_, T_4_, T_5_, and T_6_. The Tukey HSD test from three independent biological replicates (*n* = 3) shows that means (±SD) in the same graph followed by letters are substantially different at *p* < 0.05.

		Na⁺ (Shoot)	Na⁺ (Root)	K⁺ (Shoot)	K⁺ (Root)	Na⁺/K⁺ (Shoot)	Na⁺/K⁺ (Root)
T_1_	Yukinkomai	7.153 ± 0.307 ab	6.443 ± 1.821 ab	36.999 ± 0.320 a	9.979 ± 3.646 ab	0.193 ± 0.007 ab	0.756 ± 0.486 ab
	YNU31-2-4	4.015 ± 0.461 a	5.815 ± 0.209 a	73.580 ± 10.387 bc	9.540 ± 2.286 ab	0.055 ± 0.006 a	0.638 ± 0.180 ab
	YNU SL	2.395 ± 0.623 a	4.278 ± 0.475 a	103.244 ± 4.470 d	19.745 ± 3.525 bce	0.024 ± 0.007 a	0.224 ± 0.065 a
T_2_	Yukinkomai	52.891 ± 3.861 e	21.774 ± 3.349 c	39.277 ± 7.142 a	58.186 ± 6.054 fg	1.370 ± 0.204 h	0.374 ± 0.042 a
	YNU31-2-4	38.461 ± 1.138 de	12.693 ± 1.506 ac	101.262 ± 11.89 d	25.517 ± 1.060 ce	0.383 ± 0.035 bc	0.496 ± 0.039 a
	YNU SL	46.925 ± 0.740 e	14.504 ± 2.699 ac	59.984 ± 8.697 ab	33.589 ± 0.869 e	0.793 ± 0.115 d	0.431 ± 0.076 a
T_3_	Yukinkomai	173.428 ± 10.181 g	42.970 ± 2.422 d	163.663 ± 20.843 gh	14.358 ± 1.173 acd	1.074 ± 0.185 fg	3.008 ± 0.330 d
	YNU31-2-4	131.200 ± 13.936 f	17.729 ± 0.633 bc	237.981 ± 2.467 i	26.135 ± 3.356 ce	0.551 ± 0.062 cd	0.687 ± 0.104 ab
	YNU SL	259.658 ± 12.609 j	38.881 ± 1.899 d	215.377 ± 9.604 i	17.085 ± 2.165 acd	1.209 ± 0.112 gh	2.301 ± 0.323 cd
T_4_	Yukinkomai	24.048 ± 3.714 bcd	14.335 ± 0.665 ac	165.506 ± 2.965 gh	54.866 ± 12.976 f	0.145 ± 0.024 ab	0.270 ± 0.055 a
	YNU31-2-4	18.563 ± 2.238 ac	6.056 ± 1.404 c	185.275 ± 8.845 h	158.934 ± 10.385 h	0.100 ± 0.015 a	0.038 ± 0.008 a
	YNU SL	34.999 ± 3.478 ce	14.026 ± 1.653 ac	162.721 ± 1.295 gh	69.164 ± 3.512 g	0.215 ± 0.023 ab	0.203 ± 0.015 a
T_5_	Yukinkomai	2.655 ± 0.138 a	6.403 ± 0.243 ab	100.987 ± 5.386 cd	14.312 ± 2.986 acd	0.026 ± 0.000 a	0.459 ± 0.086 a
	YNU31-2-4	2.129 ± 0.216 a	6.159 ± 3.117 ab	108.075 ± 4.447 de	28.459 ± 0.918 de	0.020 ± 0.002 a	0.215 ± 0.102 a
	YNU SL	3.720 ± 0.494 a	7.716 ± 1.824 ab	124.550 ± 3.831 de	4.760 ± 0.505 a	0.030 ± 0.003 a	1.626 ± 0.367 bc
T_6_	Yukinkomai	256.632 ± 3.638 j	35.668 ± 3.350 g	132.577 ± 7.727 ef	5.065 ± 0.248 a	1.939 ± 0.084 i	7.062 ± 0.886 e
	YNU31-2-4	198.885 ± 8.74 h	26.292 ± 3.969 e	155.934 ± 8.892 fg	12.507 ± 1.528 ac	1.280 ± 0.127 gh	2.140 ± 0.503 cd
	YNU SL	235.424 ± 10.79 i	55.548 ± 9.217 f	268.199 ± 15.224 j	20.706 ± 1.664 bce	0.880 ± 0.075 ef	2.718 ± 0.674 d
