# Supplementary material for: Tolerance with High Yield Potential Is Provided by Lower Na+ Ion Accumulation and Higher Photosynthetic Activity in Tolerant YNU31-2-4 Rice Genotype under Salinity and Multiple Heat and Salinity Stress
Source: Plants (Basel). 2023 May 8;12(9):1910. doi: 10.3390/plants12091910 (PMC10180928; doi:10.3390/plants12091910)
Supplement: Supplementary file 1 [file plants-12-01910-s001.zip › Table S1.pdf]

Table S1. Variance analysis tables.

1000-Grain Weight (TG)

	Df	Sum Sq	Mean Sq	F value	Pr (>F)	
G	2	219.8	109.88	262.2	< 2e-16	***
E	5	388.4	77.69	262.2	< 2e-16	***
G x E	10	47.8	4.78	11.4	1.83E-08	***
Residuals	36	15.1	0.42			

The Net Photosynthesis Ratio (An)

	Df	Sum Sq	Mean Sq	F value	Pr (>F)	
G	2	67	33.6	6.27	0.00461	**
E	5	1069	213.9	39.9	1.04E-13	***
G x E	10	5331	533.1	99.47	< 2e-16	***
Residuals	36	193	5.4			

Ascorbate Peroxidase (APX) Activity

	Df	Sum Sq	Mean Sq	F value	Pr (>F)	
G	2	55078	27539	63.02	1.74E-12	***
E	5	568277	113655	260.08	< 2e-16	***
G x E	10	375582	37558	85.94	< 2e-16	***
Residuals	36	15732	437			

Catalase (CAT) Activity

	Df	Sum Sq	Mean Sq	F value	Pr (>F)	
G	2	510.1	255.05	108.25	5.92E-16	***
E	5	1056.9	211.38	89.72	< 2e-16	***
G x E	10	2033.5	203.35	86.31	< 2e-16	***
Residuals	36	84.8	2.36			

Chalky Grain (CG)

	Df	Sum Sq	Mean Sq	F value	Pr (>F)	
G	2	3557	1778.6	53.65	1.59E-11	***
E	5	4540	908	27.39	2.46E-11	***
G x E	10	7218	721.8	21.77	2.23E-12	***
Residuals	36	1193	33.2			

Chlorophyll a (Chla) Content

	Df	Sum Sq	Mean Sq	F value	Pr (>F)	
G	2	11758	5879	98.61	2.48E-15	***
E	5	29542	5908	99.1	< 2e-16	***
G x E	10	15180	1518	25.46	2.09E-13	***
Residuals	36	2146	60			

Chlorophyll b (Chlb) Content

	Df	Sum Sq	Mean Sq	F value	Pr (>F)	
G	2	34009	17004	49.95	4.12E-11	***
E	5	149365	29873	87.76	< 2e-16	***
G x E	10	55823	5582	16.4	1.36E-10	***
Residuals	36	12255	340			

Total Chlorophyll (ChlT) Content

	Df	Sum Sq	Mean Sq	F value	Pr (>F)	
G	2	25595	12798	49.36	4.82E-11	***
E	5	112905	22581	87.1	< 2e-16	***
G x E	10	42166	4217	16.26	1.53E-10	***
Residuals	36	9333	259			

The intercellular CO2 concentration (Ci)

	Df	Sum Sq	Mean Sq	F value	Pr (>F)	
G	2	3096	1548	8.922	7.13E-04	***
E	5	283030	56606	326.264	< 2e-16	***
G x E	10	41519	4152	23.931	5.39E-13	***
Residuals	36	6246	173			

The ratio of intercellular to ambient CO2 concentration (CiCa)

	Df	Sum Sq	Mean Sq	F value	Pr (>F)	
G	2	0.1615	0.0807	68.77	5.06E-13	***
E	5	2.0818	0.4164	354.65	< 2e-16	***
G x E	10	0.4286	0.0429	36.51	7.34E-16	***
Residuals	36	0.0423	0.0012			

ns: non-significant; **significant at P < 0.005; ***significant at P < 0.001.

Transpiration Rate (E)

	Df	Sum Sq	Mean Sq	F value	Pr (>F)	
G	2	6.40E-07	3.20E-07	0.779	4.67E-01	ns
E	5	2.65E-04	5.31E-05	129.182	< 2e-16	***
G x E	10	4.79E-05	4.79E-06	11.667	1.35E-08	***
Residuals	36	1.48E-05	4.10E-07			

Flag Leaf Area (FLA)

	Df	Sum Sq	Mean Sq	F value	Pr (>F)	
G	2	2363	1181.4	109.53	4.94E-16	***
E	5	5574	1114.8	103.35	< 2e-16	***
G x E	10	1307	130.7	12.11	8.32E-09	***
Residuals	36	388	10.8			

Grain Length (GL)

	Df	Sum Sq	Mean Sq	F value	Pr (>F)	
G	2	0.8106	0.4053	184	< 2e-16	***
E	5	2.0207	0.4041	183.48	< 2e-16	***
G x E	10	0.5038	0.0504	22.87	1.07E-12	***
Residuals	36	0.0793	0.0022			

Grain Number Per Panicle (GNPP)

	Df	Sum Sq	Mean Sq	F value	Pr (>F)	
G	2	3122	1.56E+03	65.39	<2e-16	***
E	5	67960	1.36E+04	569.33	<2e-16	***
G x E	10	4604	4.60E+02	19.29	<2e-16	***
Residuals	72	1719	2.40E+01			

Stomatal Conductance (Gs)

	Df	Sum Sq	Mean Sq	F value	Pr (>F)	
G	2	0.0279	0.01397	19.63	1.72E-06	***
E	5	0.8301	0.16602	233.24	< 2e-16	***
G x E	10	0.3914	0.03914	54.99	< 2e-16	***
Residuals	36	0.0256	0.00071			

Grain Thickness (GT)

	Df	Sum Sq	Mean Sq	F value	Pr (>F)	
G	2	0.27583	0.13791	259.49	< 2e-16	***
E	5	0.25343	0.05069	95.37	< 2e-16	***
G x E	10	0.06153	0.00615	11.58	1.50E-08	***
Residuals	36	0.01913	0.00053			

Glucose Content

	Df	Sum Sq	Mean Sq	F value	Pr (>F)	
G	2	841	420	17.9	4.01E-06	***
E	5	72487	14497	617.32	<2e-16	***
G x E	10	10406	1041	44.31	<2e-16	***
Residuals	36	845	23			

Grain Width (GW)

	Df	Sum Sq	Mean Sq	F value	Pr (>F)	
G	2	1.1248	0.5624	448.6	< 2e-16	***
E	5	1.1988	0.2398	191.25	< 2e-16	***
G x E	10	0.2893	0.0289	23.08	9.32E-13	***
Residuals	36	0.0451	0.0013			

K^+ content – Root (KR)

	Df	Sum Sq	Mean Sq	F value	Pr (>F)	
G	2	3363	1681	77.5	9.01E-14	***
E	5	45777	9155	422	< 2e-16	***
G x E	10	19116	1912	88.11	< 2e-16	***
Residuals	36	781	22			

K^+ content – Shoot (KS)

	Df	Sum Sq	Mean Sq	F value	Pr (>F)	
G	2	23669	11835	146.77	< 2e-16	***
E	5	163193	32639	404.77	< 2e-16	***
G x E	10	30947	3095	38.38	3.28E-16	***
Residuals	36	2903	81			

Malondialdehyde (MDA) Content

	Df	Sum Sq	Mean Sq	F value	Pr (>F)	
G	2	4.51	2.253	2.921	6.67E-02	ns
E	5	80.44	16.089	20.859	9.73E-10	***
G x E	10	105.07	10.507	13.623	1.76E-09	***
Residuals	36	27.77	0.771			

Na^+ Content – Root (NaR)

	Df	Sum Sq	Mean Sq	F value	Pr (>F)	
G	2	2656	1328	92.48	6.55E-15	***
E	5	353347	70669	4920.4	< 2e-16	***
G x E	10	3847	385	26.78	9.62E-14	***
Residuals	36	517	14			

Na^+ Content – Shoot (NaS)

	Df	Sum Sq	Mean Sq	F value	Pr (>F)	
G	2	9286	4643	117.45	<2e-16	***
E	5	449619	89924	2274.79	<2e-16	***
G x E	10	22329	2233	56.48	<2e-16	***
Residuals	36	1423	40			

Na^+/K^+ Ratio – Root (NaKR)

	Df	Sum Sq	Mean Sq	F value	Pr (>F)	
G	2	14.99	7.496	62.63	1.89E-12	***
E	5	94.7	18.94	158.26	< 2e-16	***
G x E	10	40.93	4.093	34.2	2.08E-15	***
Residuals	36	4.31	0.12			

Na^+/K^+ Ratio – Shoot (NaKS)

	Df	Sum Sq	Mean Sq	F value	Pr (>F)	
G	2	1.449	0.7247	95.48	4.04E-15	***
E	5	13.974	2.7949	368.23	< 2e-16	***
G x E	10	2.536	0.2536	33.41	3.02E-15	***
Residuals	36	0.273	0.0076			

Panicle Length (PL)

	Df	Sum Sq	Mean Sq	F value	Pr (>F)	
G	2	106.7	53.33	237.47	<2e-16	***
E	5	603.4	120.69	537.37	<2e-16	***
G x E	10	73.5	7.35	32.74	<2e-16	***
Residuals	72	16.2	0.22			

Panicle Number (PN)

	Df	Sum Sq	Mean Sq	F value	Pr (>F)	
G	2	273.4	136.7	31.42	1.55E-10	***
E	5	3131.4	626.3	143.97	< 2e-16	***
G x E	10	454	45.4	10.44	1.19E-10	***
Residuals	72	313.2	4.4			

Perfect Grain (PG)

	Df	Sum Sq	Mean Sq	F value	Pr (>F)	
G	2	672	336.2	26.45	8.56E-08	***
E	5	9964	1992.9	156.8	< 2e-16	***
G x E	10	4883	488.3	38.42	3.23E-16	***
Residuals	72	458	12.7			

Plant Biomass (PB)

	Df	Sum Sq	Mean Sq	F value	Pr (>F)	
G	2	16302	8151	448.2	<2e-16	***
E	5	96889	19378	1065.51	<2e-16	***
G x E	10	10622	1062	58.41	<2e-16	***
Residuals	72	1309	18			

Plant Height (PH)

	Df	Sum Sq	Mean Sq	F value	Pr (>F)	
G	2	6615	3308	205.89	< 2e-16	***
E	5	7615	1523	94.81	< 2e-16	***
G x E	10	1733	173	10.79	6.18E-11	***
Residuals	72	1157	16			

ns: non-significant; **significant at P < 0.005; ***significant at P < 0.001.

Proline (PRO) Content

	Df	Sum Sq	Mean Sq	F value	Pr (>F)	
G	2	0.897	0.449	39.1	9.44E-10	***
E	5	20.282	4.056	353.5	< 2e-16	***
G x E	10	12.153	1.215	105.9	< 2e-16	***
Residuals	36	0.413	0.011			

Protein (PROT) Concentration

	Df	Sum Sq	Mean Sq	F value	Pr (>F)	
G	2	740748	370374	47.75	7.47E-11	***
E	5	1703113	340623	43.91	2.45E-14	***
G x E	10	1683197	168320	21.7	2.35E-12	***
Residuals	36	279255	7757			

Root Biomass (RB)

	Df	Sum Sq	Mean Sq	F value	Pr (>F)	
G	2	655.2	327.6	115.909	< 2e-16	***
E	5	2591.8	518.4	183.392	< 2e-16	***
G x E	10	115.4	11.5	4.082	1.81E-04	***
Residuals	72	203.5	2.8			

Root Length (RL)

	Df	Sum Sq	Mean Sq	F value	Pr (>F)	
G	2	802.2	401.1	94.2	< 2e-16	***
E	5	1137	227.4	53.41	< 2e-16	***
G x E	10	399.8	40	9.39	9.08E-10	***
Residuals	72	306.6	4.3			

Relative Water Content (RWC)

	Df	Sum Sq	Mean Sq	F value	Pr (>F)	
G	2	610	305	9.165	6.06E-04	***
E	5	2494	498.8	14.99	5.82E-08	***
G x E	10	599.1	59.9	1.8	9.61E-02	ns
Residuals	72	1197.9	33.3			

Superoxide Dismutase (SOD) Activity

	Df	Sum Sq	Mean Sq	F value	Pr (>F)	
G	2	1513	756.6	145.6	<2e-16	***
E	5	4858	971.6	187	<2e-16	***
G x E	10	5493	549.3	105.7	<2e-16	ns
Residuals	72	187	5.2			

Spikelet Number (SN)

	Df	Sum Sq	Mean Sq	F value	Pr (>F)	
G	2	1502	751	21.492	4.80E-08	***
E	5	32859	6572	188.06	< 2e-16	***
G x E	10	2619	262	7.495	4.82E-08	***
Residuals	72	2516	35			

Water Use Efficiency (WUE)

	Df	Sum Sq	Mean Sq	F value	Pr (>F)	
G	2	6815402	3407701	3.832	3.10E-02	*
E	5	261016322	52203264	58.699	2.65E-16	***
G x E	10	39487541	3948754	4.44	4.25E-04	***
Residuals	36	32015942	889332			

Yield Per Plant (YPP)

	Df	Sum Sq	Mean Sq	F value	Pr (>F)	
G	2	962	481	89.31	<2e-16	***
E	5	22195	4439	824.56	<2e-16	***
G x E	10	1669	167	31	<2e-16	***
Residuals	72	388	5			
