# Supplementary material for: Tolerance with High Yield Potential Is Provided by Lower Na+ Ion Accumulation and Higher Photosynthetic Activity in Tolerant YNU31-2-4 Rice Genotype under Salinity and Multiple Heat and Salinity Stress
Source: Plants (Basel). 2023 May 8;12(9):1910. doi: 10.3390/plants12091910 (PMC10180928; doi:10.3390/plants12091910)
Supplement: Supplementary file 1 [file plants-12-01910-s001.zip › Table S2.pdf]

Table S2. Loading values and percentage contribution of variables on the axis identified by the principal component analysis (PCA) for all cultivars under control and saline conditions.

Variable	Dim. 1			Dim. 2			Dim. 3		
	Loading values	Contribution of variable (%)	Quality of representation	Loading values	Contribution of variable (%)	Quality of representation	Loading values	Contribution of variable (%)	Quality of representation
An	0.14	0.099	0.02	0.62	9.906	0.384	-0.009	0.003	0
Gs	0.553	1.541	0.305	0.532	7.302	0.283	-0.347	4.573	0.121
Ci	0.712	2.561	0.507	0.38	3.727	0.145	-0.258	2.524	0.067
E	0.77	2.994	0.593	0.153	0.6	0.023	-0.186	1.319	0.035
Ci.Ca	0.636	2.043	0.405	0.257	1.698	0.066	-0.627	14.892	0.393
WUE	-0.802	3.244	0.643	0.084	0.181	0.007	0.105	0.42	0.011
RWC	0.804	3.26	0.646	0.108	0.3	0.012	-0.032	0.039	0.001
Chla	0.831	3.482	0.69	0.238	1.464	0.057	0.141	0.758	0.02
Chlb	0.881	3.922	0.777	-0.116	0.348	0.013	0.096	0.351	0.009
ChlT	0.891	4.009	0.794	-0.071	0.129	0.005	0.093	0.331	0.009
PROT	0.703	2.498	0.495	-0.2	1.028	0.04	0.234	2.072	0.055
MDA	0.051	0.013	0.003	0.072	0.133	0.005	-0.043	0.072	0.002
PRO	-0.556	1.561	0.309	-0.222	1.274	0.049	-0.141	0.758	0.02
CAT	0.039	0.008	0.002	0.328	2.769	0.107	0.278	2.931	0.077
SOD	-0.211	0.226	0.045	-0.513	6.77	0.263	-0.364	5.012	0.132
APX	-0.073	0.027	0.005	-0.589	8.95	0.347	0.048	0.086	0.002
NaS	-0.866	3.79	0.751	-0.382	3.754	0.146	0.144	0.788	0.021
NaR	-0.91	4.177	0.827	-0.277	1.974	0.077	-0.051	0.098	0.003
KS	-0.593	1.778	0.352	-0.228	1.335	0.052	0.514	10.008	0.264
KR	0.056	0.016	0.003	0.493	6.257	0.243	0.501	9.529	0.251
NaKS	-0.766	2.965	0.587	-0.092	0.216	0.008	-0.053	0.106	0.003
NaKR	-0.606	1.852	0.367	-0.455	5.326	0.207	-0.019	0.013	0
PH	0.776	3.039	0.602	-0.418	4.507	0.175	0.321	3.906	0.103
PB	0.94	4.462	0.884	-0.212	1.155	0.045	-0.051	0.1	0.003
RL	0.741	2.774	0.549	0.019	0.01	0	0.203	1.57	0.041
RB	0.867	3.792	0.751	-0.381	3.737	0.145	-0.017	0.011	0
PN	0.763	2.943	0.583	0.494	6.294	0.244	0.029	0.033	0.001
PL	0.921	4.279	0.848	-0.296	2.263	0.088	0.003	0	0
FLA	0.845	3.608	0.715	-0.404	4.216	0.164	0.122	0.563	0.015
GNPP	0.899	4.078	0.808	-0.306	2.408	0.093	-0.172	1.125	0.03
SN	0.794	3.18	0.63	-0.453	5.3	0.206	-0.204	1.583	0.042
TGW	0.922	4.291	0.85	-0.098	0.249	0.01	0.181	1.239	0.033
YPP	0.928	4.352	0.862	-0.229	1.347	0.052	-0.156	0.925	0.024
PG	0.522	1.376	0.273	-0.027	0.019	0.001	-0.476	8.581	0.226
CG	0.092	0.043	0.009	0.308	2.446	0.095	0.535	10.846	0.286
GL	0.933	4.395	0.871	-0.117	0.352	0.014	0.101	0.384	0.01
GW	0.891	4.009	0.794	-0.056	0.081	0.003	0.288	3.148	0.083
GT	0.81	3.313	0.656	-0.083	0.177	0.007	0.495	9.304	0.245
Eigen value		19.807			3.88			2.637	
Variability (%)		52.124			10.211			6.94	
Cumulative (%)		52.124			62.335			69.275	
