# Supplementary figures and images for: Tolerance with High Yield Potential Is Provided by Lower Na+ Ion Accumulation and Higher Photosynthetic Activity in Tolerant YNU31-2-4 Rice Genotype under Salinity and Multiple Heat and Salinity Stress
Source: Plants (Basel). 2023 May 8;12(9):1910. doi: 10.3390/plants12091910 (PMC10180928; doi:10.3390/plants12091910)

■ Control (26°C, 0 mM NaCl)    ■ Heat (31°C, 0 mM NaCl)  
■ Salinity (26°C, 75 mM NaCl)    ■ Heat + Salinity (31°C, 75 mM NaCl)

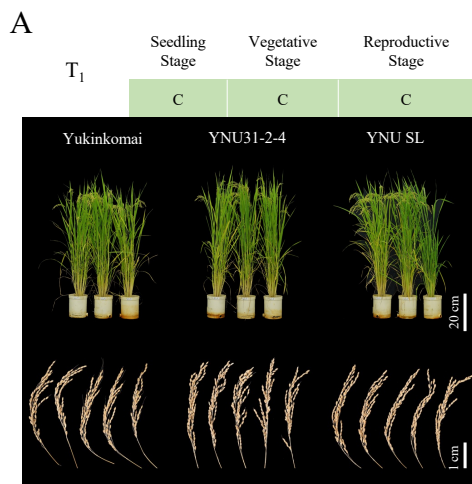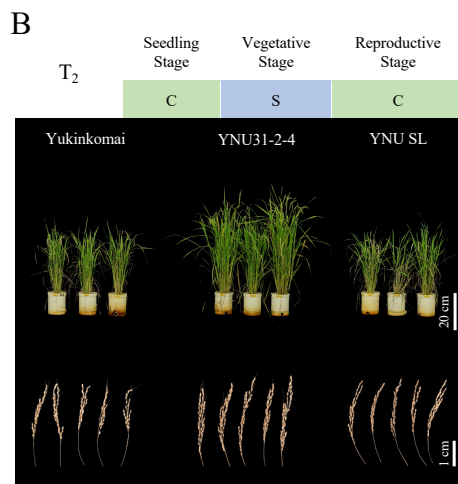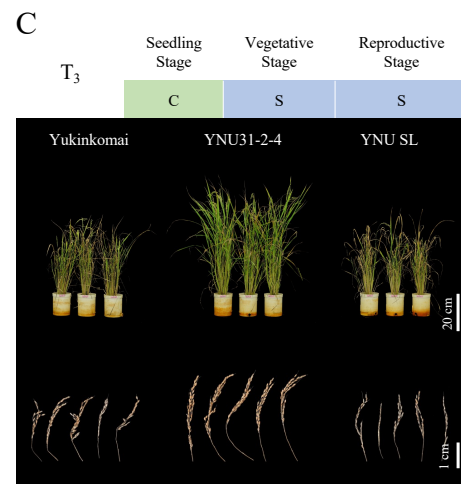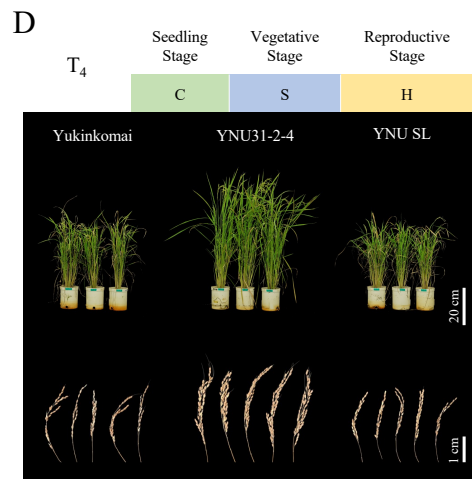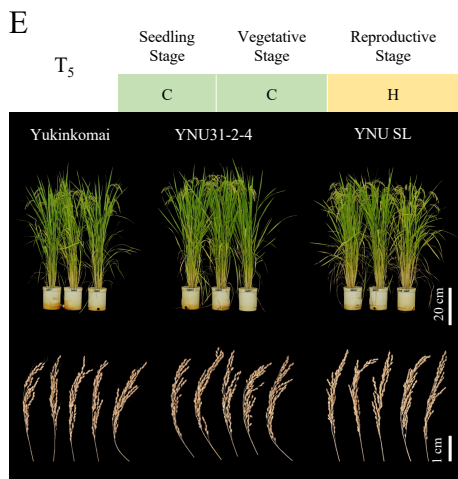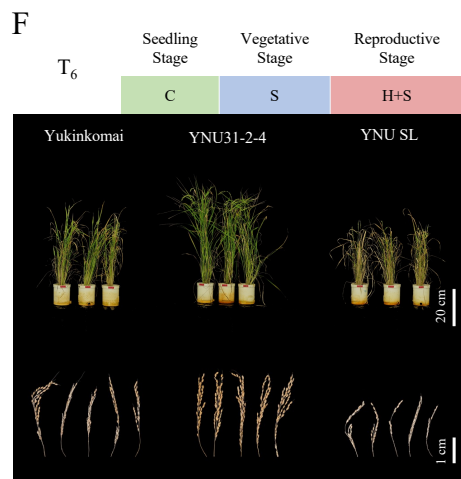

Supplement: Supplementary file 1 [file plants-12-01910-s001.zip › Figure S1_V1.pdf]
